# Determinants of Infant Growth in a Birth Cohort in the Nepal Plains

**DOI:** 10.1111/mcn.70004

**Published:** 2025-02-26

**Authors:** Laura Busert‐Sebela, Mario Cortina‐Borja, Vikas Paudel, Delanjathan Devakumar, Jonathan C. K. Wells, Dharma S. Manandhar, Naomi M. Saville

**Affiliations:** ^1^ Great Ormond Street Institute of Child Health University College London London UK; ^2^ Mother and Infant Research Activities Kathmandu Nepal; ^3^ Institute for Global Health University College London London UK

**Keywords:** adolescent pregnancy, food insecurity, low birth weight, maternal educational status, Nepal, season, stunting

## Abstract

This study aimed to identify the determinants of infant growth in terms of length‐for‐age *z*‐score (LAZ) in a birth cohort (*n* = 602) in the plains of Nepal. Children were enrolled within 72 h of birth and followed‐up every 28 days until they were 2 years. We fitted mixed‐effects linear regression models controlling for multiple measurements within individuals to examine the impact of household and maternal factors, feeding practices and infection on infant LAZ. We conducted separate analyses for the age periods 0–6 months (exclusive breastfeeding period) and 7–24 months (complementary feeding period) to check whether the importance of determinants differed by child age. Maternal factors related to both the environment in‐utero and in postnatal life were the most important determinants of infant growth. The overall most important determinant of growth was low birthweight. At birth, babies born with low birthweight had a −1.21 (−1.38, −1.05) lower LAZ compared to normal birthweight babies. The difference in LAZ between low birthweight and normal birthweight babies attenuated with age but low birthweight remained the factor with the largest effect size. The second largest factor was maternal education. Infants of mothers with any level of education had a 0.22 (0.07, 0.38) higher LAZ in the 7–24 months age‐period than those whose mothers had never been to school. Other relevant determinants were adolescent pregnancy, minimum dietary diversity, symptoms of respiratory infection, household food insecurity, season and maternal absence. The importance of maternal factors for infant growth calls for public health interventions targeting girls and young women.

## Summary

- Infant growth faltering remains a public health problem in rural Nepal. At age 2 years, half of the cohort were stunted.
- Maternal factors related to both the environment in‐utero and in postnatal life were the most important determinants of infant growth.
- The by far most important determinant of infant growth was low birthweight. Other important maternal factors were education and adolescent pregnancy.
- These findings call for public health interventions targeting girls and young women.

## Graphical abstract

This study aimed to identify the determinants of infant growth in terms of length‐for‐age *z*‐score (LAZ) in a birth cohort (*n* = 602) in the plains of Nepal. We found that maternal factors related to both the environment in‐utero and in postnatal life were the most important determinants of infant growth.

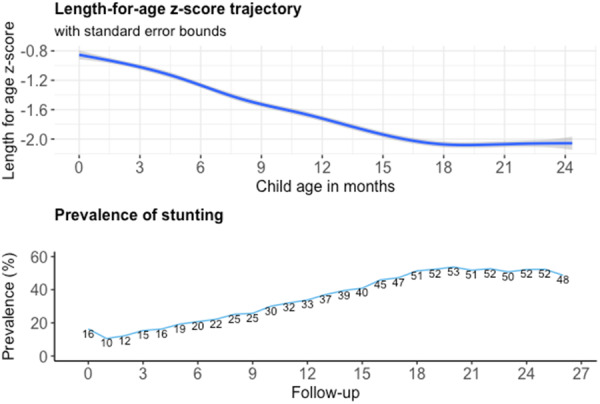

## 1 Introduction

In low‐income settings, linear growth is an overall marker of children's well‐being and indicates the extent to which their needs for overall care, adequate nutrition, stimulation and hygiene are fulfilled. Poor child growth is therefore indicative of living conditions that do not allow children to thrive and develop to their full potential (de Onis and Branca 2016). Infancy and the prenatal period are periods of rapid growth and development but are also the time when children are most sensitive to insults that could limit their growth and development. The first 1000 days of life, from conception to 2 years, are thus considered an important window of opportunity in which interventions to improve breastfeeding, complementary feeding, hygiene, protection from infectious diseases, childcare and stimulation are expected to be most effective (Martorell 2017).

Child growth faltering or stunting, defined as length/height more than two standard deviations below the age‐sex specific median of a healthy reference population, is associated with important long‐term consequences. Stunted children are more likely to have reduced motor development and cognition in childhood (Sudfeld et al. 2015), and lower earnings in adulthood (Dewey and Begum 2011; Stewart et al. 2013), though the causality of these associations remains uncertain (Leroy and Frongillo 2019). Child growth faltering is a marker of a deficient environment for healthy growth and development. Stunted children are likely to grow up to be short adults (Adair et al. 2013) and for women short stature increases the risk of adverse perinatal outcomes such as intrauterine growth restriction (IUGR) (Dewey and Begum 2011). Short mothers with a narrow pelvis are also more likely to have obstructed labour which greatly increases the risk of perinatal mortality and birth asphyxia (Dewey and Begum 2011).

The potential causes of child growth faltering occur in nearly all aspects of infant life, from the maternal environment in utero, to breastfeeding and complementary feeding practices, infection, stimulation, hygiene, and general care at home. The WHO conceptual framework on Childhood Stunting (Stewart et al. 2013, own adaptation in Supporting Information: Figure 1) comprehensively summarises these potential direct causes and embeds them in the upstream contextual factors that enable deficient environments for child growth and development. Which causal factors are most relevant is context‐dependent and the relative importance of each is likely to differ between populations and settings. The overall aim of this study was to establish, which factors contribute to child stunting in a rural population in Nepal, with high levels of child undernutrition.

Our study cohort is based in Dhanusha district, in Madesh Pradesh in the lowlands of Nepal, bordering India. This population is characterised by high levels of poverty, very low rates of formal education and, at the time of the study conception, low toilet ownership. It is a patriarchal, patrilocal society where women become a part of the husband's family and household after marriage, usually at a young age, and soon after marriage have their first child.

Using the causal factors listed in the WHO framework (Stewart et al. 2013), the objective of this study was to establish the most important determinants of infant growth in terms of length‐for‐age *z*‐score (LAZ) in the Growth Monitoring Study (GMS) cohort in Dhanusha district, Nepal. Such findings can help inform future programmes to tailor their interventions to the needs specific to this population.

## 2 Methods

### 2.1 Participants and Recruitment

The GMS was a prospective cohort study located in the plains (*Terai*) of Nepal. A STROBE checklist is provided in Supporting Information: File 1. Research ethics approval was granted by the Nepal Health Research Council (Reg. no. 95/2013). Of the then 101 geopolitical units (VDCs) in Dhanusha district, 60 were randomly selected. Incentivised local women informed data collectors about births in their community. A baby was recruited into the study if all inclusion criteria were fulfilled: (I) the mother planned to live in one of the study clusters for the next 12 months, (II) the child was a singleton baby and (III) the baby was measured within 72 h of birth. Between June and August 2012, birth measurements of 697 babies were taken, of which 602 fulfilled all inclusion criteria and were enrolled into the study.

### 2.2 Measurements

Infant length was measured in duplicate in 28 day‐intervals using a ShorrBoard stadiometer (Maryland, USA) accurate to 1 mm. On every third visit, data on infant and young child feeding practices, child morbidity, hygiene and care practices, child morbidity and care during illness were collected. The data collection was terminated once the child was 24 months of age, so that each child has up to 28 measurements. Maternal height was measured at a 6‐year follow‐up of the cohort.

### 2.3 Selection of Potential Determinants

We used the causes listed in the “WHO conceptual framework on Childhood Stunting” as described in Stewart et al. (2013) to select potential determinants of growth. Factors that are listed in the framework but are not mentioned in this study could not be considered due to a lack of data. We also considered additional factors if they were identified as relevant determinants of growth in other studies (Dorsey et al. 2018; Kramer et al. 2016; MAL‐ED Network Investigators 2017; Saville et al. 2021, 2022). A detailed description of the potential determinants and their rationale is provided in Supporting Information: File 2.

### 2.4 Statistical Methods

Analyses were restricted to children under 25 months and LAZ was the outcome of interest. We separated analyses by child age: (1) 0–6 months, corresponding to the exclusive breastfeeding period and (2) 7–24 months, corresponding to the complementary feeding period.

We visually summarised the cohort's growth over the study period in terms of prevalence of stunting and the mean LAZ trajectory. We provide summary statistics of all potential determinants that were considered in the analysis. To better understand the relationships between determinants and the nature of deprivation in this population, we additionally analysed the associations between all time‐invariant determinants considered in this analysis using chi‐squared tests, and cross‐tabulated selected associations that were considered useful in the context of this study and population.

We fitted mixed‐effects linear regression models with random effects in the intercept only (R library nlme, Pinheiro et al. 2018), which allowed for the LAZ values at birth (intercept) to vary across children, while keeping the effect of other predictors fixed. We used the Bayesian information criterion (BIC) (Kuha 2004) to assess goodness of fit and benchmark for the retention of variables in the final models. We used BIC to assess goodness of fit as its penalty term for including covariates in a model is larger than, for instance, AIC's. This results in a protective effect against overfitting, and this is an important aspect in our model selection procedure as we aim to focus only on the most important determinants of child growth in this population.

To develop the model, we created a subset of data with complete observations. We did not include maternal height at this point because it had a higher proportion of missing values and would have considerably reduced the number of observations in the model‐building data set. In a first step, we fitted the function for age using natural cubic splines, which allow for generating smooth, interpretable, and flexible covariates that can capture nonlinear patterns in nonparametric regression models (function ns from R library splines, R Core Team 2022). In a second step we conducted a forward selection procedure over potential determinants of growth using univariable analyses: Each factor was individually added to the age‐only model, and we checked whether it improved the model fit (Models 1). For each relevant indicator we also checked for interactions with child age or sex. In a third step we conducted backwards selection: we checked for collinearity using variance inflation factors between the selected indicators and removed those that contributed the least to improving goodness of fit. Models 2 included the sets of indicators with the best model fit at the respective age‐period using the subsets of data with complete observations in the selected variables.

As a final analytical step, we imputed missing values (R library mice, van Buuren and Groothuis‐Oudshoorn 2011) and repeated the analysis using the same model specifications as in Models 2 and additionally included maternal height (Models 3).

## 3 Results

### 3.1 Missing Observations

Each of the 602 children had up to 28 observations over the 2‐year study period (median 25 observations), eight in the 0–6 months age‐period, 20 in the 7–24 months period. There was a total of 13,896 observations with a valid LAZ value. Of the 4216 observations with valid LAZ in the 0–6 months age‐period, 279 (7%) had missing values in respiratory infection. Of the 9511 observations with valid LAZ in the 7–24 months period, 472 (5%) had missing values in dietary diversity and 468 (5%) in maternal absence and feeding arrangement. Birthweight was missing in six (1%) of the 602 children enrolled. Maternal height was measured at the 6‐year follow‐up and therefore had a higher proportion of missing at 13% (77). In the 0–6 months age‐period, 3907 of 4216 (93%) had complete covariates, in the 7–24 months age‐period 8080 of 9511 (85%).

Children whose mothers' height could not be measured, had lower LAZ in infancy, even after controlling for other covariates, thus we could not assume that missingness occurred at random. We therefore report the results from the complete cases (Models 2) as the main results. However, the estimates from the complete cases (Models 2) and those from multiple imputations (MI, Models 3) were very similar and where differences occurred this was likely attributable to the fact that the models derived from MI (Models 3) included maternal height while the models with complete cases (Models 2) did not. For that reason, we report both the results from the complete cases models and the MI models where the estimates differ by 0.04 LAZ or more.

### 3.2 Growth Trajectory and Independent Variables

**Figure 1:**
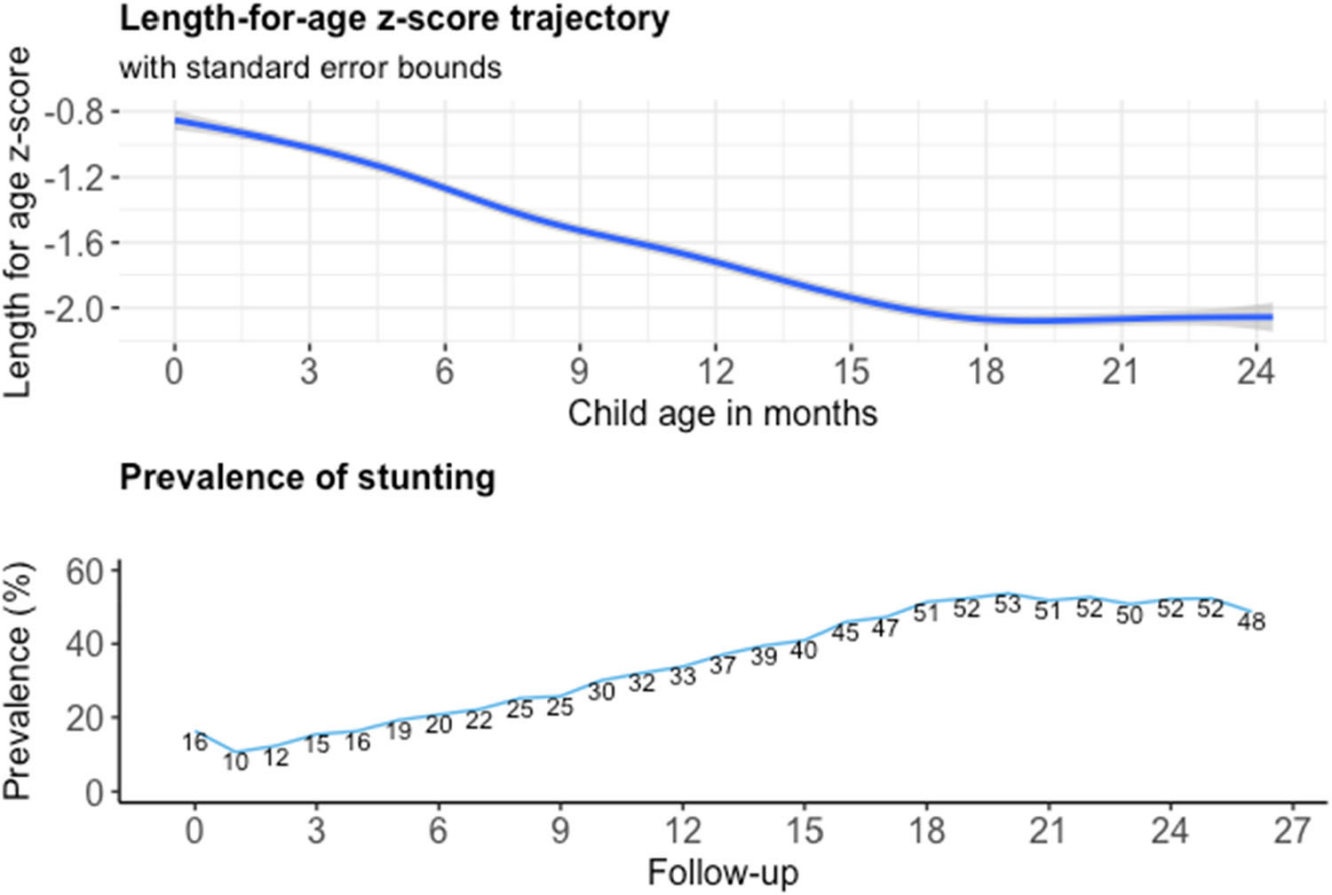
Length‐for‐age *z*‐score and stunting prevalence in the Growth Monitoring Study (GMS) cohort.

The growth trajectory in terms of stunting prevalence and LAZ is presented in Figure 1. The prevalence of stunting was highest at 2 years when around half of the cohort were stunted. All the potential determinants of infant growth that were considered in this study are described in Tables 1 and 2. Table 1 presents the baseline characteristics. Nearly one‐third of the children (31%) had low birthweight and only 33% of mothers had ever been to school. One in five mothers were adolescent at the time of the study child's birth. Most of the households practised open defecation (78%). Time‐varying independent variables are presented in Table 2. Minimum dietary diversity was very low initially (4% at follow‐up 9) but increased somewhat as the children aged (33% at follow‐up 24). The associations between determinants are presented in Supporting Information: File 3. We found that despite adolescent mothers being less likely to be from the poorest asset quartile and more likely to have some formal education, they were more likely to have a low birthweight baby. Mothers who had been to school were more likely to initiate breastfeeding within 1 h of giving birth, more likely to come from households with their own water source and a toilet, but surprisingly were also more likely to be food insecure. The prevalence of low birthweight babies was lower among women from households in the better off asset quartile.

**Table 1:** Baseline characteristics of the GMS cohort^a^.

Baseline characteristics	% (*n*)
Sex of study child	
Boy	52% (314)
Girl	48% (288)
Household factors	
Asset quartile	
1 (worse off)	25% (150)
2	25% (150)
3	25% (150)
4 (better off)	25% (150)
Household experiences has any degree of food insecurity	29% (177)
Water source	
Public/neighbours well, pump or tap	29% (176)
Own pump/well/tap/borehole	71% (426)
Household uses open defecation	78% (467)
Number of older siblings	
0	31% (186)
1	20% (120)
2 or more	49% (296)
Maternal factors	
Low birthweight	31% (185)
Birth‐to‐pregnancy‐interval before study child	
Primigravida	26% (159)
< 24 months	30% (181)
≥ 24 months	30% (178)
End date of previous pregnancy unknown	14% (84)
Mother adolescent (≤ 19 years) at study child's birth	20% (123)
Mother has any level of education	33% (198)
Mother ate less, more or the same amount during the last trimester of pregnancy	
Less	36% (217)
Same	57% (342)
More	7% (43)
Maternal height (cm), mean (SD)	150.9 (5.5)
Breastfeeding practices	
Breastfeeding initiated withing 1 h after birth	27% (163)
Colostrum was discarded^a^	22% (135)
Total	602

Abbreviations: GMS, Growth Monitoring Study; SD, standard deviation.

^a^ Missing observations: Low birthweight: 6, asset quartile: 2, colostrum: 2, maternal height: 77.

**Table 2:** Time‐varying independent variables in the GMS: Childcare, infection and feeding.

	3	6	9	12	15	18	21	24
Follow‐up number	*n* = 494	*n* = 530	*n* = 505	*n* = 503	*n* = 501	*n* = 506	*n* = 514	*n* = 489
Mother's absence and care arrangement								
Doesn't work outside, takes break to feed the baby or takes baby with her	93% (459)	92% (485)	84% (425)	80% (404)	76% (381)	75% (377)	75% (386)	73% (355)
Baby is with another carer	2% (11)	2% (12)	6% (30)	8% (41)	10% (52)	12% (60)	10% (53)	14% (70)
No feeding arrangement	5% (24)	6% (33)	10% (50)	11% (55)	13% (63)	12% (63)	13% (69)	11% (53)
Missing	0% (0)	0% (0)	0% (0)	1% (3)	1% (5)	1% (6)	1% (6)	2% (11)
Diarrhoea (14 days recall)								
No diarrhoea	91% (452)	83% (442)	50% (251)	67% (335)	80% (399)	73% (371)	76% (391)	68% (334)
Child had diarrhoea	9% (42)	17% (88)	50% (254)	33% (168)	19% (97)	26% (130)	23% (116)	29% (144)
Missing	0% (0)	0% (0)	0% (0)	0% (0)	1% (5)	1% (5)	1% (7)	2% (11)
Cough with fast breathing (14 days recall)								
No cough and fast breathing	66% (324)	66% (348)	77% (388)	85% (429)	86% (430)	91% (458)	87% (449)	86% (422)
Child had cough with fast breathing	34% (170)	34% (182)	23% (117)	15% (74)	13% (66)	8% (43)	11% (58)	11% (55)
Missing	0% (0)	0% (0)	0% (0)	0% (0)	1% (5)	1% (5)	1% (7)	2% (12)
Continued breastfeeding								
Stopped breastfeeding	0% (0)	0% (2)	0% (1)	1% (3)	3% (13)	4% (22)	7% (37)	15% (73)
Continued breastfeeding at that timepoint	100% (494)	100% (528)	100% (504)	99% (500)	97% (487)	96% (484)	92% (475)	83% (406)
Missing	0% (0)	0% (0)	0% (0)	0% (0)	0% (1)	0% (0)	0% (2)	2% (10)
Exclusive breastfeeding (24 h recall)								
Child not exclusively breastfed	26% (130)	35% (186)						
Child exclusively breastfed	74% (364)	65% (344)						
Missing	0% (0)	0% (0)						
Minimum dietary diversity (24 h recall)								
No minimum dietary diversity (< 4/7 food groups)			96% (487)	80% (403)	82% (411)	78% (395)	70% (359)	64% (313)
Minimum dietary diversity (≥ 4/7 food groups)			4% (18)	20% (100)	17% (85)	21% (106)	29% (149)	33% (163)
Missing			0% (0)	0% (0)	1% (5)	1% (5)	1% (6)	3% (13)
Minimum meal frequency (24 h recall)								
No minimum meal frequency			58% (295)	45% (224)	27% (137)	25% (125)	21% (110)	20% (98)
Minimum meal frequency			32% (163)	47% (234)	71% (354)	72% (362)	77% (396)	77% (378)
Missing			9% (47)	9% (45)	2% (10)	4% (19)	2% (8)	3% (13)

Abbreviation: GMS, Growth Monitoring Study.

### 3.3 Determinants of LAZ

**Figure 2:**
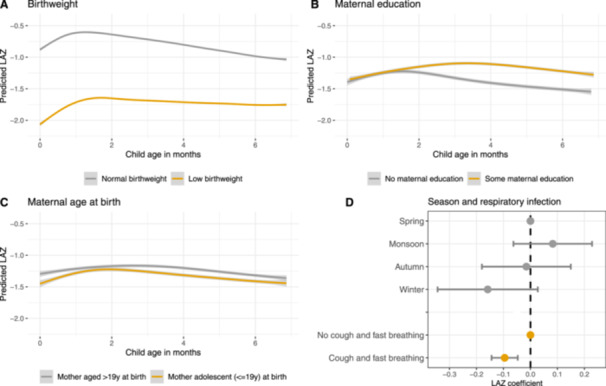
Summary of determinants of infant growth 0–6 months as predicted from the regression results. Determinants with age‐interactions (low birthweight, maternal education and maternal age at birth) are presented as trajectories of predicted length‐for‐age *z*‐score (with standard errors) (A–C). Determinants with main effects only (season, cough with fast breathing) are presented in a coefficient plot (D). LAZ, length‐for‐age *z*‐score.

**Figure 3:**
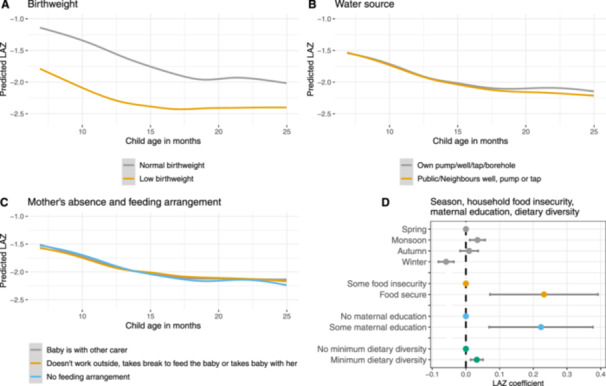
Summary of determinants of infant growth 7–24 months as predicted from the regression results. Determinants with age‐interactions (low birthweight, water source, maternal absence and feeding arrangements) are presented as trajectories of predicted length‐for‐age *z*‐score (with standard errors) (A–C). Determinants with main effects only (season, food insecurity, maternal education, and minimum dietary diversity) are presented in a coefficient plot (D). LAZ, length‐for‐age *z*‐score.

The final model for the age‐period from 0 to 6 months included low birthweight, maternal education, and maternal age at birth with age‐interactions, and season, respiratory infection and maternal height with main effects only (no age‐interactions). The final model for the age‐period 7–24 months included low birthweight, water source, and maternal absence for work and feeding arrangements with age‐interactions, and season, maternal education, minimum dietary diversity, household food insecurity and maternal height as main effects only. The full regression results are provided in Supporting Information: File 4. For ease of reading, only the results of the final models (Models 2 with complete cases) are presented in Figure 2 for the age‐period from 0 to 6 months and in Figure 3 for the age‐period 7–24 months. The effects of determinants with age‐interactions are presented as predicted LAZ trajectories, the effects of determinants with main effects only are presented as coefficient plots.

### 3.4 Maternal Factors

Low birthweight was by far the most important determinant of infant growth in both growth periods. At birth, low birthweight babies had −1.21 (−1.38, −1.05) lower LAZ than normal birthweight babies. In both age‐periods, the effect of low birthweight attenuated with age, with low birthweight babies faltering at a lower rate (Figures 2A and 3A). At 7 months, the effect size had decreased, and low birthweight babies were −0.75 (−0.91, −0.59) LAZ shorter than normal birthweight babies, but birthweight remained the determinant with the largest effect size. In the models using MI which include maternal height, the effect size was somewhat smaller with low birthweight babies being −1.13 (−1.29, −0.96) and −0.63 (−0.78, −0.48) LAZ shorter in the 0–6 months and 7–24 months age‐period, respectively.

Maternal education was a relevant determinant in both age‐periods, but the effect changed over time. At birth, there was no difference, but during the first 6 months of life babies of mothers with at least some education grew better (Figure 2B). In the 7–24 months age‐period, the effect of maternal education did not vary by child age and after adjustment children of mothers with education had a 0.22 (0.07, 0.38) higher LAZ than those of mothers without any education (Figure 3D). In the model using MI including maternal height, the effect size was smaller with 0.18 (0.03, 0.32) LAZ.

In the first 6 months of life, babies of adolescent mothers were smaller than those of mothers who were 20 years or older at the time of birth, but the effect varied with child age. At birth, children of adolescent mothers had a −0.22 (−0.41, −0.03) lower LAZ, after adjustment for covariates, but caught up some of the difference between 1 and 2 months. After this, the trajectories diverged a little and tracked alongside until age 6 months when the difference disappeared (Figure 2C). In the model using MI and including maternal height, the size of the main effect was larger, and the babies of adolescent mothers were −0.26 (−0.44, −0.07) LAZ shorter, but the age‐interaction remained the same. In the 7–24 months age‐period we observed no difference.

In the first 6 months age‐period we found no difference in LAZ by mother's absence from home for work and their baby feeding arrangements. In the age‐period 7–24 months there were some, albeit very small, differences, especially around 18 months when children whose mothers worked outside the home but had not made feeding arrangements had a lower LAZ than children whose mothers did not work outside the home, took their child with them, fed the child during breaks, or left it with another carer (Figure 3C).

Maternal height (in cm) was a determinant of infant growth in both the 0–6 months age‐period (0.04 [0.02, 0.05]) and the 7–24 months age‐period (0.06 [0.05, 0.07]).

Maternal factors that were not included in the final model were eating behaviour in the last trimester (did not improve the model fit) and birth‐to‐pregnancy interval (caused multicollinearity).

### 3.5 Home Environment

Household food insecurity was a determinant of infant growth in the 7–24 months age‐period. After adjustment, children from food secure households had a 0.23 (0.07, 0.39) higher LAZ than children in households with some level of food insecurity (Figure 3D). This effect size was smaller with 0.16 (0.01, 0.31) LAZ in the model from MI including maternal height.

The water source was also included in the final model with an age‐interaction in the 7–24 months age‐period. Initially, the predicted LAZ trajectories of children from households with their own water source and those who used a public or a neighbour's well, pump or tap were the same, but from around 18 months the trajectories diverged with children from households with their own water source having a slightly higher LAZ (Figure 3B).

Factors in the home environment that were not included in the final model were presence of older siblings (caused multicollinearity), household wealth and toilet use (did not improve the model fit).

### 3.6 Breastfeeding

None of the breastfeeding indicators improved the model fit and were therefore not included in the final model.

### 3.7 Inadequate Complementary Feeding

Minimum dietary diversity had a positive, albeit very small, effect on LAZ in the 7–24 months age‐period. Children who ate from at least four out of seven food groups had a 0.03 (0.01, 0.05) higher LAZ than those who ate from only three or less food groups (Figure 3D). Continued breastfeeding and minimum meal frequency did not improve the model fit and were therefore not retained in the analysis.

### 3.8 Infection

In the 0–6 months age‐period, children with symptoms of respiratory infection in the 2 weeks preceding the survey had a −0.10 (−0.14, −0.05) lower LAZ than those with no symptoms (Figure 2D). We found no differences by respiratory infection in the 7–24 months period, or by diarrhoea.

### 3.9 Other Factors

In the 7–24 months age‐period, using spring as the reference category, children were 0.03 (0.01, 0.06) LAZ taller in monsoon and 0.06 (−0.08, −0.04) LAZ shorter in winter (Figure 3D). We found no difference by child sex as a main effect or as an interaction with other covariates.

## 4 Discussion

### 4.1 Summary and Discussion of Main Findings

The objective of this analysis was to establish the main determinants of infant growth in a birth cohort in Dhanusha district, Nepal. We found that maternal factors were the most important predictors of infant LAZ. Most importantly, low birthweight as an indicator of IUGR had the largest effect size and even after controlling for maternal height children born with a birthweight of less than 2500 g were more than one LAZ smaller than those with normal birthweight. Although this difference attenuated over the 2 years, children born with low birthweight tracked far below that of children with normal birthweight. While the association between birthweight and length of the baby is by no means surprising since weight and size are closely associated, this finding is indicative of the importance of the prenatal period and the maternal environment for healthy growth and development, and corroborates previous studies (Christian et al. 2013; MAL‐ED Network Investigators 2017).

Maternal age at birth, specifically being adolescent, was a further maternal factor that determined the growth trajectory in the first 6 months. Babies born to mothers 19 years or younger were smaller at birth and continued to track below the growth trajectory of those babies born to older mothers, but differences did not persist beyond 6 months. Similarly, a joint analysis of birth cohort studies from five countries found that children born to adolescent mothers were shorter at 2 years (Fall et al. 2015). In an Indian study, adolescent pregnancy was associated with child growth through lower levels of education, lower socioeconomic status and poorer nutritional status among adolescent mothers (Nguyen et al. 2019). In the present cohort, however, adolescent mothers were more likely to have attended school and less likely to be among the poorest asset quartile. These better nonbiological drivers of child growth among adolescent mothers could explain the catch‐up in growth over the first 6 months of life. The smaller size at birth in babies born to adolescent mothers may be explained by the competition for resources between the growing mother and the growing foetus (Scholl et al. 1994). Some of the effect of maternal age might also partly be explained by the fact that most—but sadly not all—of the adolescent mothers were primigravidae and the babies of primiparous women tend to be smaller than those of later births (Billewicz and Thomson 1973).

Another maternal factor that determined infant growth throughout the first 2 years of life is education. At birth, we found no difference between children of mothers who had ever been to school, but from 2 months onwards, the LAZ of children whose mothers had attended school declined at a lower rate than of those whose mothers had never been to school, and the difference persisted until the end of the study. A similar pattern was found in a pooled analysis of 33 birth cohort studies where LAZ did not differ by maternal education at birth but became important over the child's first 2 years of life (Mertens et al. 2022). Using data from the same area in Nepal to study the association between age at marriage and child growth, and adjusting for maternal education, Wells et al. (2022) found that babies whose mothers had at least secondary education had lower odds of stunting than those of mothers without any education at age 6 to 12 months, but this was not found amongst neonates. Another study in the same area as the GMS found that children of mothers with secondary education were taller than those of mothers with no education (Devakumar et al. 2017). A study from Nepal using nationally representative data found maternal education to be inversely associated with the risk of stunting (Dorsey et al. 2018). Using data from four rounds of Demographic and Health Surveys (DHS, 1996–2011), authors found maternal education to be one of the main drivers of improvements in child linear growth in Nepal (Cunningham et al. 2017). A similar analysis using an additional round of DHS (2016) corroborated these findings (Hanley‐Cook et al. 2022). The mechanism through which maternal education affects infant growth is likely through better knowledge about care and feeding practices.

We observed no difference in LAZ by maternal absence and feeding arrangement in the first 6 months, but a difference, albeit very small, at around 18 months. There are various possible explanations for the lack of association in the first 6 months and the very small difference in later life. First, the number of mothers working away from home was very small at the younger ages (7% and 8% at follow‐up three and six, respectively). Second, the indicator does not differentiate by the duration of absence and includes a wide range, between 1h and 9 h per day. Third, it is unclear how children were cared for and what they were fed when they were with other carers. The age when children who were left at home without feeding arrangements while their mother worked away from home had a noticeably lower LAZ than the reference group (around 18 months) coincided with the seasons autumn and winter, which is the transition time between the lean season and harvest (Saville et al. 2021). Indeed, the duration of absence of those mothers who did work outside the home was highest at follow‐up 18 (mean age 16.7 months) with 3.3 h per day compared with 2.1–2.8 h at the other occasions, probably due to the higher workload during harvest season. It is possible that food scarcity and prolonged duration of mother's absence without any feeding arrangements explains the slight drop in LAZ at this age.

Season was included in the final models in both age‐periods, but only in the later period (7–24 months) was there a significant difference in LAZ between seasons. Children had the lowest LAZ in winter and the highest in monsoon. This finding is consistent with that of Saville et al. (2021) in the same region of Nepal where authors found neonates to be shortest in winter and longest during the hot season. Additionally, they found a second peak in LAZ during spring which we did not observe in this cohort. In this population, the winter season is preceded by the lean postmonsoon season (Saville et al. 2021) and it is possible that there is a lagged effect of food scarcity on growth in height. This explanation was offered by other authors who observed similar patterns. A pooled analysis using data of eight cohort studies found that wasting or declines in weight‐for‐length *z*‐score (WLZ) were followed by lower LAZ, and authors mentioned seasonality in agriculture or infectious diseases as one potential driver behind the variability in WLZ (Richard et al. 2012).

Children with symptoms of respiratory infection had a 0.1 lower LAZ than children without symptoms in the first 6 months of life, but there was no effect in the later age‐period. A pooled analysis of seven cohort studies, including from Nepal, found no association between symptoms of acute lower respiratory infection and growth in under 2‐year‐old children (MAL‐ED Network Investigators 2017). The observed effect at 0–6 months might be explained by a loss of appetite during infection, or a diversion of energy away from growth towards recovery.

There was only a very small difference in growth by minimum dietary diversity. On a conceptual level this is surprising as inadequate dietary intake is listed among the proximal determinants of child undernutrition (Black et al. 2008), but this finding is in line with previous studies that consistently show positive, but often small, effects of dietary diversity on child growth. In Nepal, a decomposition analysis using nationally representative data found that minimum dietary diversity only made a small contribution to the improvements in HAZ from 2001 to 2016 (Hanley‐Cook et al. 2020). One reason for the small effect size may be the indicator used. A dietary diversity score is only a rough approximation of the probability of micronutrient adequacy (Verger et al. 2021) and does not consider the quantity of food eaten from a food group since even a small amount consumed is enough to be included in the score.

Household food insecurity was negatively associated with a LAZ in children aged 7–24 months, possibly due to micronutrient deficiencies resulting from reduced consumption of nutrient‐rich expensive foods in times of financial strain (Iannotti et al. 2012), or macronutrient deficiency due to a reduction of overall food intake. The evidence from other studies in Nepal has been mixed, with some showing no association (Busert et al. 2016; Dorsey et al. 2018; Osei et al. 2010) and others a higher risk of stunting in food insecure households (Paudel et al. 2013; Singh et al. 2014). It is possible that intra‐household food allocation changes in times of food scarcity (Harris‐Fry et al. 2017) and offers some degree of protection for children at the expense of adults.

Children living in households with a drinking water source on their own premises grew better than those whose household relied on the neighbour's or a public water source, but this effect only appeared after 15 months. This indicator is unlikely to signal water quality but rather ease of access to water. It is likely that families who do not have a water source on their own premises find it more difficult to follow good hygiene practices such as frequent handwashing, cleanliness around the preparation of foods and disposal of baby stools. Poor access to water and sanitation is associated with environmental enteric dysfunction (EED), an acquired enteropathy of the small intestine which has been associated with stunting (Tickell et al. 2019).

Toilet use did not improve the model fit and was therefore not included in the final model. This contrasts with the findings from an analysis of DHS data where the drop in open defecation was identified as one of the drivers behind the improvements in LAZ in Nepal (Cunningham et al. 2017; Headey and Hoddinott 2015). The authors, however, used open defecation at the community‐level rather than at the household level, arguing that open defecation is a negative externality because household members are largely immune to much of their own bacteria (Headey and Hoddinott 2015; Spears 2013). At the time of this study, the prevalence of open defecation was very high in this population at 78%, so that even those families who did use toilets will have been exposed to the faeces and bacteria of their neighbours.

Diarrhoea was not associated with LAZ in either age‐period. Like the present study, an analysis of seven birth cohort studies found no effect of diarrhoea on growth but showed that the presence of enteropathogens in non‐diarrhoeal stools increased the risk of stunting in the first 24 months (MAL‐ED Network Investigators 2017). The role of subclinical inflammation and EED in the aetiology of child stunting has gained increased attention but could not be addressed in the study because of a lack of data.

In summary, the present study corroborates the central role of maternal factors in the aetiology of child stunting.

### 4.2 Study Strengths and Limitations

This analysis has several major strengths. The longitudinal design with many observations per child allowed a precise modelling of child growth. The wealth of data available allowed us to consider many potential covariates, many of which were measured repeatedly, and to include factors that have received little attention in previous studies, such as maternal absence for work. Levels of missing data were low and were addressed with MI. Another important strength of our analysis is that we tested every potential covariate for age‐interactions and were thus able to identify determinants that would have been overlooked had we only considered main effects. For example, the effect of water source only became apparent after including an age‐interaction. Examining time‐varying effects can reveal more specific target groups and important windows for intervention.

Our study is also subject to some limitations that need to be considered in the interpretation of its results. Only singleton births were included in this study so the results may not be fully applicable to twins/multiple births. Another limitation relates to our use of the term “determinant” which could be understood to imply causality. As with all observational research, this study cannot ascertain whether all the identified risk factors are causal or merely correlates A third limitation relates to the generalisability of our findings. While we believe these to be applicable to other Maithili‐speaking populations in Nepal and north India, they may not be fully generalisable to other geographical regions and may also have reduced validity in the future. Many determinants are shaped by culture and evolve over time, so their relevance in the context of infant growth faltering may also change. As an example, at a 6‐year follow‐up of this cohort, the prevalence of toilet use had increased dramatically to 67% of households.

## 5 Conclusion

This study echoes previous research that emphasized the role of maternal factors and the prenatal environment in infant growth (Danaei et al. 2016; MAL‐ED Network Investigators 2017; Mertens et al. 2022; Wells et al. 2022). These findings call for public health interventions targeting girls and young women with the aim of preventing early marriage, improving nutrition at pre‐conception, during pregnancy, and beyond.

In Nepal, where women typically get married before getting pregnant, interventions aimed at preventing early marriage are key in the prevention of child growth faltering. While the implications of underage marriage go far beyond child growth faltering (Marphatia et al. 2017) and is ultimately a human rights issue, we want to briefly highlight how delaying marriage and consequently first pregnancy until adulthood is vital in the context of child malnutrition. First, early marriage typically has negative consequences for girls' education in South Asia (Marphatia et al. 2017) and as this study has shown, poor education in turn is negatively associated with child growth. Second, education is likely to have a positive effect on women's empowerment and bargaining power which have been shown to be positively associated with child growth in Nepal (Cunningham et al. 2015; Kulkarni et al. 2021). Third, preventing adolescent pregnancy is likely to benefit offspring growth, as this study has shown. Lastly, a study in this population in Nepal has demonstrated that both early marriage and early pregnancy are independently associated with shorter maternal adult height (Marphatia et al. 2021). As this study and previous research consistently has shown, maternal height is an important predictor of child growth. Child marriage is illegal in Nepal but this law has gaps and is only very weakly enforced (Human Rights Watch 2016). This is attested by the very high prevalence of child marriage in this cohort where 81% of mothers were married before the age of 18.

Interventions in South Asia have shown that improving the women's nutrition during pregnancy and pre‐conception can have positive effects on the infants' birthweight (Chowdhury et al. 2022; Diamond‐Smith et al. 2022; Saville et al. 2018). These interventions often involved mothers‐in‐law and husbands to get their support, enable the purchase of more nutritious food, and improve intra‐household food allocation in favour of the woman. This emphasis on the importance the prenatal period and mothers should not imply a diversion of attention away from the postnatal period. Complementary feeding interventions and nutrition education have been shown to have positive, albeit small, effects on child linear growth (Lassi et al. 2020; Panjwani and Heidkamp 2017) and deserve increased attention.

## Author Contributions

Vikas Paudel and Naomi M. Saville designed the research study, Vikas Paudel, Naomi M. Saville and Dharma S. Manandhar conducted the study, Laura Busert‐Sebela analysed the data and wrote the paper, Mario Cortina‐Borja advised on the analysis, Jonathan C. K. Wells and Delanjathan Devakumar commented on the manuscript.

## Ethics Statement

Ethical approval was granted by the Nepal Health Research Council (Reg. no. 95/2013).

## Conflicts of Interest

The authors declare no conflicts of interest.

## Supporting information

Supporting information.

Supporting information.

Supporting information.

Supporting information.

## Data Availability

The data that support the findings of this study are available online at the UCL Research Data Repository. Growth Monitoring Study (GMS) dataset for the analysis on the determinants of infant growth in a birth cohort in the Nepal plains (Saville et al. 2024).
