# Supplementary material for: Determinants of Infant Growth in a Birth Cohort in the Nepal Plains
Source: Matern Child Nutr. 2025 Feb 26;21(3):e70004. doi: 10.1111/mcn.70004 (PMC12150145; doi:10.1111/mcn.70004)
Supplement: Supplementary file 1 — Supporting information. [file MCN-21-e70004-s001.doc]

**Supplementary File 1: STROBE Statement**

STROBE Statement—Checklist of items that should be included in reports of ***cohort studies***

|  | Item No | Recommendation |
| --- | --- | --- |
| **Title and abstract** | 1 | *(*a) Indicate the study’s design with a commonly used term in the title or the abstract  “This study aimed to identify the determinants of infant growth in terms of length-for-age z-score (LAZ) in a birth cohort (*n*=602) in the plains of Nepal.” |
|  |  | (*b*) Provide in the abstract an informative and balanced summary of what was done and what was found  “This study aimed to identify the determinants of infant growth in terms of length-for-age z-score (LAZ) in a birth cohort (*n*=602) in the plains of Nepal. Children were enrolled within 72 hours of birth and followed-up every 28 days until they were 2 years. We used mixed-effects linear regression controlling for multiple measurements within individuals to examine the impact of household and maternal factors, feeding practices and infection on infant LAZ. We conducted separate analyses for the age periods 0-6 months (exclusive breastfeeding period) and 7-24 months (complementary feeding period) to check whether the importance of determinants differed by child age. Maternal factors related to both the environment in-utero and in postnatal life were the most important determinants of infant growth.” |
| Introduction | | |
| Background/rationale | 2 | Explain the scientific background and rationale for the investigation being reported  “In low-income settings, linear growth is an overall marker of children’s well-being and indicates the extent to which their needs for overall care, adequate nutrition, stimulation, and hygiene are fulfilled. Poor child growth is therefore indicative of living conditions that do not allow children to thrive and develop to their full potential (de Onis & Branca, 2016). Infancy and the prenatal period are periods of rapid growth and development but are also the time when children are most sensitive to insults that could limit their growth and development. The first 1000 days of life, from conception to two years, are thus considered an important window of opportunity in which interventions to improve breastfeeding, complementary feeding, hygiene, protection from infectious diseases, childcare and stimulation are expected to be most effective (Martorell, 2017).  Child growth faltering or stunting, defined as length/height more than two standard deviations below the age-sex specific median of a healthy reference population, is associated with important long-term consequences. Stunted children are more likely to have reduced motor development and cognition in childhood (Sudfeld et al., 2015), and lower earnings in adulthood (Dewey & Begum, 2011; Stewart, Iannotti, Dewey, Michaelsen, & Onyango, 2013), though the causality of these associations remains uncertain (Leroy & Frongillo, 2019). Child growth faltering is a marker of a deficient environment for healthy growth and development. Stunted children are likely to grow up to be short adults (Adair et al., 2013) and for women short stature increases the risk of adverse perinatal outcomes such as intrauterine growth restriction (IUGR) (Dewey & Begum, 2011). Short mothers with a narrow pelvis are also more likely to have obstructed labour which greatly increases the risk of perinatal mortality and birth asphyxia (Dewey & Begum, 2011).  The potential causes of child growth faltering occur in nearly all aspects of infant life, from the maternal environment in utero, to breastfeeding and complementary feeding practices, infection, stimulation, hygiene, and general care at home. The WHO conceptual framework on Childhood Stunting (Stewart et al., 2013, own adaptation in Supplementary Figure 1) comprehensively summarises these potential direct causes and embeds them in the upstream contextual factors that enable deficient environments for child growth and development. Which causal factors are most relevant is context-dependent and the relative importance of each is likely to differ between populations and settings.” |
| Objectives | 3 | State specific objectives, including any prespecified hypotheses  “Using the causal factors listed in the WHO framework (Stewart et al., 2013), the objective of this study was to establish the most important determinants of infant growth in terms of length-for-age z-score (LAZ) in the Growth Monitoring Study (GMS) cohort in Dhanusha district, Nepal.” |
| Methods | | |
| Study design | 4 | Present key elements of study design early in the paper  The GMS was a prospective cohort study located in the plains (*Terai*) of Nepal |
| Setting | 5 | Describe the setting, locations, and relevant dates, including periods of recruitment, exposure, follow-up, and data collection  “Of the then 101 geopolitical units (VDCs) in Dhanusha district, 60 were randomly selected. Incentivised local women informed data collectors about births in their community. A baby was recruited into the study if all inclusion criteria were fulfilled: (I) the mother planned to live in one of the study clusters for the next 12 months, (II) the child was a singleton baby, and (III) the baby was measured within 72 hours of birth. Between June and August 2012, birth measurements of 697 babies were taken, of which 602 fulfilled all inclusion criteria and were enrolled into the study. Measurements Infant length was measured in duplicate in 28 day-intervals using a ShorrBoard stadiometer (Maryland, USA) accurate to 1mm. On every third visit, data on infant and young child feeding practices, child morbidity, hygiene and care practices, child morbidity and care during illness were collected. The data collection was terminated once the child was 24 months of age, so that each child has up to 28 measurements. Maternal height was measured at a six-year follow-up of the cohort. “ |
| Participants | 6 | (*a*) Give the eligibility criteria, and the sources and methods of selection of participants. Describe methods of follow-up  “Of the then 101 geopolitical units (VDCs) in Dhanusha district, 60 were randomly selected. Incentivised local women informed data collectors about births in their community. A baby was recruited into the study if all inclusion criteria were fulfilled: (I) the mother planned to live in one of the study clusters for the next 12 months, (II) the child was a singleton baby, and (III) the baby was measured within 72 hours of birth.” |
|  |  | (*b*) For matched studies, give matching criteria and number of exposed and unexposed  Not relevant |
| Variables | 7 | Clearly define all outcomes, exposures, predictors, potential confounders, and effect modifiers. Give diagnostic criteria, if applicable  Outcome: length-for-age Z-scores  Exposures: “We used the causes listed in the “WHO conceptual framework on Childhood Stunting” as described in Stewart et al. (2013) to select potential determinants of growth. Factors that are listed in the framework but are not mentioned in this study could not be considered due to a lack of data. We also considered additional factors if they were identified as relevant determinants of growth in other studies (Dorsey et al., 2018; Kramer, Veile, & Otárola-Castillo, 2016; MAL-ED Network Investigators, 2017; Saville et al., 2021; Saville et al., 2022). A detailed description of the potential determinants and their rationale is provided in Supplementary File 2” |
| Data sources/ measurement | 8* | For each variable of interest, give sources of data and details of methods of assessment (measurement). Describe comparability of assessment methods if there is more than one group  Please see Supplementary File 2 |
| Bias | 9 | Describe any efforts to address potential sources of bias  Study VDCs were randomly selected.  All births in the randomly selected VDCs were considered for inclusion if they fulfilled the few inclusion criteria. |
| Study size | 10 | Explain how the study size was arrived at  Investigators aimed for a sample size of *n*=600. Using the function pwr.norm.test from library pwr in R (Champely, 2020) we calculated that with this sample size a comparison of two groups of equal size (i.e. 300 per group), under the assumption of normal distributions and equal variances, yields the power to detect a difference equal to or more than 0.162 length-for-age *z*-scores (LAZ) with power 80% and a 5% significance level. Similarly, a group size of *n*=200 has a power of 80% at 5% significance level to detect a difference of less or equal to 0.2 LAZ.   | **Sample (per group)** | **Standard deviation** | **Significance level** | **Power** | **Effect size** | | --- | --- | --- | --- | --- | | 175 | 1 | 0.05 | 0.80 | 0.212 | | 200 | 1 | 0.05 | 0.80 | 0.200 | | 225 | 1 | 0.05 | 0.80 | 0.187 | | 250 | 1 | 0.05 | 0.80 | 0.177 | | 300 | 1 | 0.05 | 0.80 | 0.162 |   (Please note that the above information is not included in the publication.) |
| Quantitative variables | 11 | Explain how quantitative variables were handled in the analyses. If applicable, describe which groupings were chosen and why  See Supplementary File 2 |
| Statistical methods | 12 | 1. Describe all statistical methods, including those used to control for confounding   “Analyses were restricted to children under 25 months and LAZ was the outcome of interest. We separated analyses by child age: 1) 0-6 months, corresponding to the exclusive breastfeeding period, and 2) 7-24 months, corresponding to the complementary feeding period.  We visually summarised the cohort’s growth over the study period in terms of prevalence of stunting and the mean LAZ trajectory. We provide summary statistics of all potential determinants that were considered in the analysis. To better understand the relationships between determinants and the nature of deprivation in this population, we additionally analysed the associations between all time-invariant determinants considered in this analysis using chi-squared tests, and cross-tabulated selected associations that were considered useful in the context of this study and population.  We fitted mixed-effects linear regression models with random effects in the intercept only (R library nlme, Pinheiro, Bates, DebRoy, Sarkar, & R Core Team, 2018), which allowed for the LAZ values at birth (intercept) to vary across children, while keeping the effect of other predictors fixed. We used the Bayesian information criterion (BIC) (Kuha, 2004) to assess goodness of fit and benchmark for the retention of variables in the final models. We used BIC to assess goodness of fit as its penalty term for including covariates in a model is larger than, for instance, AIC’s. This results in a protective effect against overfitting, and this is an important aspect in our model selection procedure as we aim to focus only on the most important determinants of child growth in this population.  To develop the model, we created a subset of data with complete observations. We did not include maternal height at this point because it had a higher proportion of missing values and would have considerably reduced the number of observations in the model-building dataset. In a first step, we fitted the function for age using natural cubic splines, which allow for generating smooth, interpretable, and flexible covariates that can capture non-linear patterns in nonparametric regression models (function ns from R library splines, R Core Team, 2022). In a second step we conducted a forward selection procedure over potential determinants of growth using univariable analyses: Each factor was individually added to the age-only model, and we checked whether it improved the model fit (Models 1). For each relevant indicator we also checked for interactions with child age or sex. In a third step we conducted backwards selection: we checked for collinearity using variance inflation factors between the selected indicators and removed those that contributed the least to improving goodness of fit. Models 2 included the sets of indicators with the best model fit at the respective age-period using the subsets of data with complete observations in the selected variables.  As a final analytical step, we imputed missing values (R library mice, van Buuren & Groothuis-Oudshoorn, 2011) and repeated the analysis using the same model specifications as in Models 2 and additionally included maternal height (Models 3).” |
|  |  | (b) Describe any methods used to examine subgroups and interactions  - “We separated analyses by child age: 1) 0-6 months, corresponding to the exclusive breastfeeding period, and 2) 7-24 months, corresponding to the complementary feeding period. “  - “For each relevant indicator we also checked for interactions with child age or sex.” |
|  |  | *(*c) Explain how missing data were addressed  “As a final analytical step, we imputed missing values using the R library mice (R library mice, van Buuren & Groothuis-Oudshoorn, 2011) and repeated the analysis using the same model specifications as in Models 2 and additionally included maternal height (Models 3).” |
|  |  | (*d*) If applicable, explain how loss to follow-up was addressed |
|  |  | (*e*) Describe any sensitivity analyses |
| Results | | |
| Participants | 13* | (a) Report numbers of individuals at each stage of study—eg numbers potentially eligible, examined for eligibility, confirmed eligible, included in the study, completing follow-up, and analysed  “Between June and August 2012, birth measurements of 697 babies were taken, of which 602 fulfilled all inclusion criteria and were enrolled into the study.“ |
|  |  | (b) Give reasons for non-participation at each stage |
|  |  | (c) Consider use of a flow diagram |
| Descriptive data | 14* | (a) Give characteristics of study participants (eg demographic, clinical, social) and information on exposures and potential confounders  The outcome LAZ is described in Figure 1, independent variables are described in Tables 1 and 2. |
|  |  | (b) Indicate number of participants with missing data for each variable of interest  “Of the 4,216 observations with valid LAZ in the 0-6 months age-period, 279 (7%) had missing values in respiratory infection. Of the 9,511 observations with valid LAZ in the 7-24 months period, 472 (5%) had missing values in dietary diversity and 468 (5%) in maternal absence and feeding arrangement. Birthweight was missing in six (1%) of the 602 children enrolled. Maternal height was measured at the six-year follow-up and therefore had a higher proportion of missing at 13% (77). In the 0-6 months age-period, 3,907 of 4,216 (93%) had complete covariates, in the 7-24 months age-period 8,080 of 9,511 (85%).” |
|  |  | (c) Summarise follow-up time (eg, average and total amount)  “Each of the 602 children had up to 28 observations over the two-year study period (median 25 observations), eight in the 0-6 months age-period, 20 in the 7-24 months period.” |
| Outcome data | 15* | Report numbers of outcome events or summary measures over time  Outcome LAZ is described in Figure 1 |
| Main results | 16 | *(*a) Give unadjusted estimates and, if applicable, confounder-adjusted estimates and their precision (eg, 95% confidence interval). Make clear which confounders were adjusted for and why they were included  All unadjusted and adjusted regression results are reported in Supplementary File 4. |
|  |  | *(*b) Report category boundaries when continuous variables were categorized  Not relevant |
|  |  | *(*c) If relevant, consider translating estimates of relative risk into absolute risk for a meaningful time period  Not relevant |
| Other analyses | 17 | Report other analyses done—eg analyses of subgroups and interactions, and sensitivity analyses  All analyses are described in 12. |
| Discussion | | |
| Key results | 18 | Summarise key results with reference to study objectives  “The objective of this analysis was to establish the main determinants of infant growth in a birth cohort in Dhanusha district, Nepal. We found that maternal factors were the most important predictors of infant LAZ. Most importantly, low birthweight as an indicator of IUGR had the largest effect size and even after controlling for maternal height children born with a birthweight of less than 2500g were more than one LAZ smaller than those with normal birthweight.” |
| Limitations | 19 | Discuss limitations of the study, taking into account sources of potential bias or imprecision. Discuss both direction and magnitude of any potential bias  “Our study is also subject to some limitations that need to be considered in the interpretation of its results. Only singleton births were included in this study so the results may not be fully applicable to twins/multiples. Another limitation relates to our use of the term “determinant” which could be understood to imply causality. As with all observational research, this study cannot ascertain whether all the identified risk factors are causal or merely correlates.” |
| Interpretation | 20 | Give a cautious overall interpretation of results considering objectives, limitations, multiplicity of analyses, results from similar studies, and other relevant evidence  “This study echoes previous research that emphasized the role of maternal factors and the prenatal environment in infant growth (Danaei et al., 2016; MAL-ED Network Investigators, 2017; Mertens et al., 2022; Wells et al., 2022). These findings call for public health interventions targeting girls and young women with the aim of preventing early marriage, improving nutrition at pre-conception, during pregnancy, and beyond.” |
| Generalisability | 21 | Discuss the generalisability (external validity) of the study results  “A third limitation relates to the generalisability of our findings. While we believe these to be applicable to other Maithili-speaking populations in Nepal and north India, they may not be fully generalisable to other geographical regions and may also have reduced validity in the future. Many determinants are shaped by culture and evolve over time, so their relevance in the context of infant growth faltering may also change. As an example, at a six-year follow-up of this cohort, the prevalence of toilet use had increased dramatically to 67% of households.” |
| Other information | | |
| Funding | 22 | Give the source of funding and the role of the funders for the present study and, if applicable, for the original study on which the present article is based  The Growth Monitoring Study (GMS) was funded by the Wellcome Trust Strategic Award for the Population Science of Maternal and Newborn Survival (grant 085417MA/Z/08/Z) and Bill and Melinda Gates Foundation (Investment ID OPP1113344). L Busert-Sebela was funded by the UCL Great Ormond Street Institute of Child Health Child Health Research PhD studentship. |

*Give information separately for exposed and unexposed groups.

**Note:** An Explanation and Elaboration article discusses each checklist item and gives methodological background and published examples of transparent reporting. The STROBE checklist is best used in conjunction with this article (freely available on the Web sites of PLoS Medicine at http://www.plosmedicine.org/, Annals of Internal Medicine at http://www.annals.org/, and Epidemiology at http://www.epidem.com/). Information on the STROBE Initiative is available at http://www.strobe-statement.org.
