# Supplementary material for: Determinants of Infant Growth in a Birth Cohort in the Nepal Plains
Source: Matern Child Nutr. 2025 Feb 26;21(3):e70004. doi: 10.1111/mcn.70004 (PMC12150145; doi:10.1111/mcn.70004)
Supplement: Supplementary file 2 — Supporting information. [file MCN-21-e70004-s003.docx]

**Supplementary File 1**


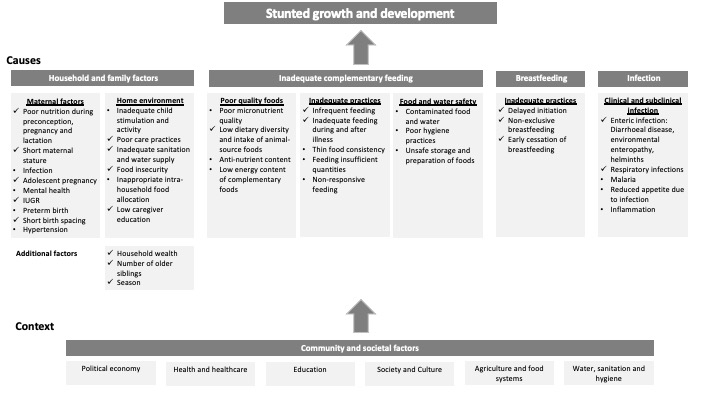


Supplementary Figure 1: Conceptual framework (own adaptation of the WHO conceptual framework on Childhood Stunting: Context, Causes and Consequences with an emphasis on complementary feeding (Stewart et al., 2013). The causes included in this analysis are marked with a✓.)

Potential determinants of infant growth: Measurement and justification

### Maternal factors

We did not have any information regarding the mother’s nutritional status or nutritional intake before pregnancy and during lactation, but shortly after birth the mothers were asked to quantify their diet in the last trimester of their pregnancy and whether they had eaten more, the same or less compared to before. A deliberate reduction of food intake during pregnancy due to cultural beliefs or fear that consumption of large amounts of food leads to a larger baby which in turn will increase the risk of a difficult birth has been mentioned in the literature. A study in Sarlahi district, nearby Dhanusha district, found this practice of “eating down” to be less common but that women often reduced food intake for other reasons such of aversion to certain foods or lack of appetite (Christian et al., 2006). Eating less during pregnancy can reduce the diet quality and maternal weight gain (Harding et al., 2017).

Maternal height was measured at the six-year follow-up and included as a continuous variable. One mother was 13 and three were 16 years at the time of the study child’s birth, and they might have continued growing until the maternal height measurement at the six-year follow-up. But for most of the mothers the measurement will reflect their height during pregnancy.

Adolescent pregnancy was defined as a mother being 19 years or younger at the time of the study child’s birth. The continued growth during adolescence competes with the nutritional demands of the growing foetus (Scholl, Hediger, Schall, Khoo, & Fischer, 1994) and can thus increase the risk of stunted growth.

The birth-to-pregnancy interval describes the time between the mother’s previous child’s birth or end of pregnancy, and conception of the study child (WHO, 2005). It was calculated by subtracting the date when the last pregnancy ended from the study child’s birth date, and then subtracting the 280 days of pregnancy. If intervals between pregnancies are short the mother’s nutrient reserves may not have enough time to replete which can have adverse effects for mother and child (Dewey & Cohen, 2007). Furthermore, an overlap between breastfeeding and pregnancy can have negative implications for the breastmilk composition and negatively affect growth of the later baby (Conde-Agudelo, Rosas-Bermúdez, Castaño, & Norton, 2012). We used the cut-off of 24 months as the recommended minimum birth-to-pregnancy interval (WHO, 2005) and added two additional categories, for primigravidae and for those mothers who did not remember the date of the end of the previous pregnancy.

We used low birthweight defined as birthweight <2500g as a measure of intrauterine growth restriction (UNICEF, 2004). Only one baby was born preterm (at seven months gestation), therefore we could not include the factor preterm birth in my analysis. We had no information regarding hypertension or infections during pregnancy.

### Home environment

Food insecurity was assessed using the FANTA Household Food Insecurity in Access category (Coates, Swindale, & Bilinsky, 2007) which captures the respondent’s perceived level of household food insecurity over the 30 days preceding the interview. The questionnaire contains nine questions which cover three domains of food insecurity: 1) anxiety and uncertainty about the household food supply, 2) insufficient quality of foods, and 3) insufficient quantity of foods and its physical consequences. The original indicator groups households into four categories: Food secure, or mildly, moderately, or severely food insecure, but because most households self-identified as food secure, we created a binary indicator by collating all degrees of food insecurity. Food insecurity limits the household’s access to sufficient amounts of high-quality foods which in turn can reduce the macro- and micronutrient intake of children.

To characterise the household’s drinking water, we distinguished between those with their own source of drinking water and those who used a public or neighbour’s source. This variable only allows limited inferences about the drinking water’s cleanliness, but the absence of a drinking water source can impede good hygiene practices which are important tools to prevent environmental enteropathy. We used a binary variable indicating whether the household used any kind of toilet or defecated in the open to describe the type of toilet facility.

Only very limited information was available regarding infant care practices. On every third visit the mother was asked whether she had been working away from the home in the past three months and how the child had been taken care of during her absence, especially with regard to feeding arrangements. We distinguished between mothers who did not work outside or took their child with them or took breaks between work to feed the baby, mothers who left the child with another carer in her absence, and mothers who did not work away from home. Especially in the exclusive breastfeeding period but also afterwards, babies need to be fed in frequent intervals and the mother’s prolonged absence may cause inadequate nutrient intake for the baby. We expected that mothers who left the baby with another carer or who made no feeding arrangement had a lower LAZ than children whose mothers did not work away from home, took the child with them, or took breaks to feed the baby, and that the effect would be larger in the exclusive breastfeeding period when babies depend on their mothers to feed them, especially in a population where expressing breastmilk and feeding infant formula are uncommon. Under the care practices domain, We also included medical care during illness, which is described in the paragraph *Infection* below.

In order to describe maternal education, we created a binary variable indicating whether the mother had ever been to school. Since the literacy rate in this population is very low, especially among women, we could not use a more granulated differentiation between levels of maternal education. Maternal education has been shown to be positively associated with child nutrition outcomes, likely due to better caring practices (Stewart, Iannotti, Dewey, Michaelsen, & Onyango, 2013). Child stimulation and intra-household food allocation could not be considered because of a lack of data.

### Inadequate Complementary Feeding and Breastfeeding

Feeding practices change over the first years of life, from the exclusive breastfeeding period to the complementary feeding period. This poses a challenge when analysing data that covers different periods, because indicators are only relevant in a certain time window and cannot be used over the whole age-range. We therefore separated the dataset into two feeding periods, the exclusive breastfeeding period from birth to six months, and the complementary feeding period between seven months and two years (the end of the monthly follow-up).

We principally used the WHO (2010) IYCF indicators as they are widely used standard indicators^[[1]](#footnote-1)^. For the exclusive breastfeeding period we used the variables Initiation of breastfeeding within one hour of birth (corresponding to IYCF indicator 1), and Exclusive breastfeeding in the first six months (IYCF indicator 2). We additionally included a variable indicating whether colostrum was fed to the new-born and not discarded, as is practised in many communities in the Hindu cultural region (Laroia & Sharma, 2006). Colostrum is important because it provides immune protection and helps to prepare the lining of the gut to receive the nutrients in milk (WHO, 2009).

In the complementary feeding period, we used the variables continued breastfeeding (corresponding to IYCF indicator 3), minimum dietary diversity (IYCF indicator 5), and minimum meal frequency (IYCF indicator 6). For the indicators regarding dietary diversity and meal frequency we also considered the dietary diversity score (range 1 to 7, WHO (2010)) and the number of feeding times per day as continuous variables, but we achieved better model fits with the binary variables.

We did not have any information regarding micronutrient intake, the amount of vitamins and minerals that the child consumes, anti-nutrient content such as phytates which inhibit mineral absorption and frequently occur in the predominantly plant-based diets in poor populations (Gibson, Bailey, Gibbs, & Ferguson, 2010), energy content, consistency and quantity of complementary foods, or whether the mother practiced responsive feeding. The factors listed under the domain “Food and water supply” also could not be considered as no data was available.

### Infection

A high burden of infectious disease early in life is frequently cited as a critical proximal cause of childhood stunting, with diarrhoeal disease possibly being among the most important factors due to its role in the malabsorption of nutrients (Black et al., 2008). Diarrhoeal disease was determined by asking the mother whether the child had had loose stools more than three times a day in the two weeks preceding the interview. For those children with diarrhoea, we further assessed whether they had been fed appropriately during the illness, defined as continued (breast-)feeding and administration of oral rehydration therapy (UNICEF, 2019). Since the binary indicator for only diarrhoea received a better fit, we did not include the indicator for appropriate feeding during illness in the analysis.

Respiratory infection was assessed by asking whether the child had been coughing combined with rapid breathing in the two weeks preceding the interview. For children who had shown symptoms of respiratory infection, we further differentiated between those who had received care by a medical professional (e.g. hospital or health post) and those who had not been presented to a medical professional or for whom medical care was only sought from an unqualified source (e.g. a Shaman, at a medical shop) (UNICEF, 2019). The binary indicator for respiratory infection achieved a better model fit so that care-seeking was not included in the final model.

Other enteric infections, malaria, reduced appetite due to infection, and inflammation could not be considered as no data were available.

### Additional factors not mentioned in the WHO framework

Although household socioeconomic status is not explicitly mentioned in the framework, we decided to include it because resource availability has been identified as an important determinant of stunting in other regions of Nepal (Dorsey et al., 2018; MAL-ED Network Investigators, 2017). We calculated an asset score using principal component analysis and included the following variables: the husband’s level of education, the materials of the house’s wall and roof, the number of bedrooms in the house, land owned, ownership of these assets in the household: electricity, radio, colour TV, bicycle, ox cart, motorbike, landline or mobile phone, pump set. Households were stratified into asset score quartiles.

Siblings can be both a hindrance and a source of support for the healthy development of a small child (Kramer, Veile, & Otárola-Castillo, 2016). When birth intervals are short, pregnancy or the birth of a sibling can displace the infant from the mother’s breast, and the baby becomes a competition for the mother’s attention and time. Older siblings can be a source of support if they are old enough to help around the house and take care of the younger children. At the same time, they can increase the younger child’s exposure to pathogens and infections, which in turn is a risk factor for growth failure. In resource-poor households, siblings may be a source of competition. We included the number of older siblings in the analysis, but no information was available regarding the birth of a younger sibling during the study period. Information on the age of older siblings was unfortunately not recorded.

In a population from this area of Nepal, seasonal patterns have been identified to be strongly correlated with maternal and newborn anthropometry and maternal dietary characteristics (Saville et al., 2021). We therefore included a categorical variable indicating in which of the four main seasons the child was measured: spring (months Chait to Jyesth in the Nepali calendar, corresponding to mid-March to mid-June), monsoon (Asad to Bhadau, mid-June to mid-September), autumn (Asoj to Mangsir, mid-September to mid-December), and winter (Paush to Phagun, mid-December to mid-March).

At the child level, we furthermore included sex as a potential risk factor because I considered preferential treatment of boys over girls plausible in this population. At the same time, the fast absolute growth rate of male foeti and infants increases their risk of stunting if their higher energy requirements cannot be met (Saville et al., 2022).

### Dealing with variables that were only measured on every third visit

Children were measured every 28 days, but potential time-varying determinants of growth such as feeding practices and infection were only measured on every third visit. To be able to use all measurement occasions we carried the value of the respective determinant forward to the next two measurement occasions when only the children’s anthropometric measurements had been recorded. For example, the value for diarrhoea at follow-up 12 was carried forward to follow-up 13 and 14. We decided to carry the values forward rather than backward because this corresponds to the chronological order in that the determinant precedes the outcome. The only exception is follow-up three when many time-varying determinants were measured for the first time and therefore had to be carried backwards to the preceding measurement occasions, too.

### References

Black, R. E., Allen, L. H., Bhutta, Z. A., Caulfield, L. E., de Onis, M., Ezzati, M., . . . Rivera, J. (2008). Maternal and child undernutrition: global and regional exposures and health consequences. *The Lancet, 371*(9608), 243-260. doi:10.1016/S0140-6736(07)61690-0

Christian, P., Bunjun Srihari, S., Thorne-Lyman, A., Khatry, S. K., Leclerq, S. C., & Ram Shrestha, S. (2006). Eating Downin Pregnancy: Exploring Food-Related Beliefs and Practices of Pregnancy in Rural Nepal. *Ecology of Food and Nutrition, 45*(4), 253-278. doi:10.1080/03670240600846336

Coates, J., Swindale, A., & Bilinsky, P. (2007). *Household Food Insecurity Access Scale (HFIAS) for Measurement of Food Access: Indicator Guide (v. 3).* Retrieved from <https://pdf.usaid.gov/pdf_docs/Pnadk896.pdf>

Conde-Agudelo, A., Rosas-Bermúdez, A., Castaño, F., & Norton, M. H. (2012). Effects of Birth Spacing on Maternal, Perinatal, Infant, and Child Health: A Systematic Review of Causal Mechanisms. *Studies in Family Planning, 43*(2), 93-114. doi:10.1111/j.1728-4465.2012.00308.x

Dewey, K. G., & Cohen, R. J. (2007). Does birth spacing affect maternal or child nutritional status? A systematic literature review. *Matern Child Nutr, 3*(3), 151-173. doi:10.1111/j.1740-8709.2007.00092.x

Dorsey, J. L., Manohar, S., Neupane, S., Shrestha, B., Klemm, R. D. W., & West, K. P. (2018). Individual, household, and community level risk factors of stunting in children younger than 5 years: Findings from a national surveillance system in Nepal. *Matern Child Nutr, 14*(1), e12434. doi:10.1111/mcn.12434

Gibson, R. S., Bailey, K. B., Gibbs, M., & Ferguson, E. L. (2010). A Review of Phytate, Iron, Zinc, and Calcium Concentrations in Plant-Based Complementary Foods Used in Low-Income Countries and Implications for Bioavailability. *Food Nutr Bull, 31*(2_suppl2), S134-S146. doi:10.1177/15648265100312s206

Harding, K. L., Matias, S. L., Mridha, M. K., Vosti, S. A., Hussain, S., Dewey, K. G., & Stewart, C. P. (2017). Eating down or simply eating less? The diet and health implications of these practices during pregnancy and postpartum in rural Bangladesh. *Public Health Nutr, 20*(11), 1928-1940. doi:10.1017/s1368980017000672

Kramer, K. L., Veile, A., & Otárola-Castillo, E. (2016). Sibling Competition & Growth Tradeoffs. Biological vs. Statistical Significance. *PLoS One, 11*(3), e0150126. doi:10.1371/journal.pone.0150126

Laroia, N., & Sharma, D. (2006). The Religious and Cultural Bases for Breastfeeding Practices Among the Hindus. *Breastfeeding Medicine, 1*(2). doi:10.1089/bfm.2006.1.94

MAL-ED Network Investigators. (2017). Childhood stunting in relation to the pre- and postnatal environment during the first 2 years of life: The MAL-ED longitudinal birth cohort study. *PLoS Med, 14*(10), e1002408. doi:10.1371/journal.pmed.1002408

Saville, N. M., Cortina-Borja, M., De Stavola, B. L., Pomeroy, E., Marphatia, A., Reid, A., . . . Wells, J. C. (2021). Comprehensive analysis of the association of seasonal variability with maternal and neonatal nutrition in lowland Nepal. *Public Health Nutr*, 1-16. doi:10.1017/s1368980021003633

Saville, N. M., Harris‐Fry, H., Marphatia, A., Reid, A., Cortina‐Borja, M., Manandhar, D. S., & Wells, J. C. (2022). Differences in maternal and early child nutritional status by offspring sex in lowland Nepal. *American Journal of Human Biology, 34*(3). doi:10.1002/ajhb.23637

Scholl, T. O., Hediger, M. L., Schall, J. I., Khoo, C. S., & Fischer, R. L. (1994). Maternal growth during pregnancy and the competition for nutrients. *Am J Clin Nutr, 60*(2), 183–188. doi:10.1093/ajcn/60.2.183

Stewart, C. P., Iannotti, L., Dewey, K. G., Michaelsen, K. F., & Onyango, A. W. (2013). Contextualising complementary feeding in a broader framework for stunting prevention. *Matern Child Nutr, 9 Suppl 2*, 27-45. doi:10.1111/mcn.12088

UNICEF. (2004). *Low birthweight. Country, regional and global estimates*. Retrieved from <https://www.unicef.org/publications/files/low_birthweight_from_EY.pdf>

UNICEF. (2019). *MICS6 Indicators and definitions*. Retrieved from <http://mics.unicef.org/tools>

WHO. (2005). *Report of a WHO Technical Consultation on Birth Spacing*. Retrieved from Geneva, Switzerland: <https://apps.who.int/iris/bitstream/handle/10665/69855/WHO_RHR_07.1_eng.pdf;sequence=1>

WHO. (2009). *Infant and young child feeding : model chapter for textbooks for medical students and allied health professionals*. Retrieved from <https://apps.who.int/iris/bitstream/handle/10665/44117/9789241597494_eng.pdf?sequence=1>

WHO. (2010). *Indicators for assessing infant and young child feeding practices part 2: measurement.* Retrieved from <https://www.who.int/maternal_child_adolescent/documents/9789241599290/en/>

WHO, & UNICEF. (2021). *Indicators for assessing infant and young child feeding practices: definitions and measurement methods.* Retrieved from <https://www.who.int/publications/i/item/9789240018389>

1. The 2008/2010 WHO/UNICEF IYCF indicators presented here were replaced in April 2021, during the writing of this thesis, by a revised set of indicators (WHO & UNICEF, 2021). I used the 2010 indicators because the data collection tools and the data analysis were designed with reference to those indicators and before the revised set of indicators had been published. [↑](#footnote-ref-1)
