# Supplementary material for: Determinants of Infant Growth in a Birth Cohort in the Nepal Plains
Source: Matern Child Nutr. 2025 Feb 26;21(3):e70004. doi: 10.1111/mcn.70004 (PMC12150145; doi:10.1111/mcn.70004)
Supplement: Supplementary file 3 — Supporting information. [file MCN-21-e70004-s002.docx]

**Supplementary File 3: Associations between potential determinants**

**Supplementary Table 3.1: Associations between potential time-invariant determinants considered in the analysis. *P*-values from Chi-squared-tests.**

|  | **Child sex** | **SES** | **Food insecurity** | **Water** | **Toilet** | **Older siblings** | **Low birth-weight** | **Maternal education** | **Birth spacing** | **Adoles-cent mother** | **Restrictive eating last trimester** | **Bf within 1hr after birth** | **Colos-trum discarded** |
| --- | --- | --- | --- | --- | --- | --- | --- | --- | --- | --- | --- | --- | --- |
| **Child sex** | NA | 0.63 | 0.35 | 0.76 | 0.68 | 0.43 | 0.04 | 0.83 | 0.43 | 0.59 | 0.38 | 0.65 | 0.44 |
| **SES** | 0.63 | NA | 0.00 | 0.00 | 0.00 | 0.73 | 0.11 | 0.00 | 0.09 | 0.03 | 0.41 | 0.00 | 0.64 |
| **Food insecurity** | 0.35 | 0.00 | NA | 0.00 | 0.00 | 0.59 | 0.78 | 0.00 | 0.05 | 0.21 | 0.42 | 0.00 | 0.32 |
| **Water** | 0.76 | 0.00 | 0.00 | NA | 0.00 | 0.01 | 0.29 | 0.01 | 0.66 | 0.15 | 0.70 | 1.00 | 0.40 |
| **Toilet** | 0.68 | 0.00 | 0.00 | 0.00 | NA | 0.96 | 0.31 | 0.00 | 0.12 | 0.34 | 0.71 | 0.00 | 0.50 |
| **Older siblings** | 0.43 | 0.73 | 0.59 | 0.01 | 0.96 | NA | 0.95 | 0.53 | 0.85 | 0.91 | 0.01 | 0.71 | 0.28 |
| **Low birthweight** | 0.04 | 0.11 | 0.78 | 0.29 | 0.31 | 0.95 | NA | 0.86 | 0.00 | 0.00 | 0.86 | 0.83 | 1.00 |
| **Maternal education** | 0.83 | 0.00 | 0.00 | 0.01 | 0.00 | 0.53 | 0.86 | NA | 0.00 | 0.03 | 0.62 | 0.00 | 0.02 |
| **Birth spacing** | 0.43 | 0.09 | 0.05 | 0.66 | 0.12 | 0.85 | 0.00 | 0.00 | NA | 0.00 | 0.38 | 0.03 | 0.44 |
| **Adolescent mother** | 0.59 | 0.03 | 0.21 | 0.15 | 0.34 | 0.91 | 0.00 | 0.03 | 0.00 | NA | 0.24 | 0.04 | 0.64 |
| **Restrictive eating last trimester** | 0.38 | 0.41 | 0.42 | 0.70 | 0.71 | 0.01 | 0.86 | 0.62 | 0.38 | 0.24 | NA | 0.19 | 0.41 |
| **Bf within 1hr after birth** | 0.65 | 0.00 | 0.00 | 1.00 | 0.00 | 0.71 | 0.83 | 0.00 | 0.03 | 0.04 | 0.19 | NA | 0.07 |
| **Colostrum discarded** | 0.44 | 0.64 | 0.32 | 0.40 | 0.50 | 0.28 | 1.00 | 0.02 | 0.44 | 0.64 | 0.41 | 0.07 | NA |

**Supplementary Table 3.2: Distribution of time-invariant determinants across selected factors (adolescent mother, maternal education, low birthweight, household asset quartile). *P*-values grom Chi-squared-tests.**

|  | Adolescent mother | | | Maternal education | | | Birthweight | | | Asset quartile | | | | |
| --- | --- | --- | --- | --- | --- | --- | --- | --- | --- | --- | --- | --- | --- | --- |
|  | >=20 years | ≤19 years | p-value | No edu | Some edu | p-value | Normal BW | Low BW | p-value | 1 | 2 | 3 | 4 | p-value |
|  | N=479 | N=123 |  | N=404 | N=198 |  | N=411 | N=185 |  | N=150 | N=150 | N=150 | N=150 |  |
| Child sex |  |  | 0.52 |  |  | 0.76 |  |  | 0.03 |  |  |  |  | 0.63 |
| boy | 53% | 50% |  | 52% | 53% |  | 55% | 45% |  | 53% | 49% | 56% | 50% |  |
| girl | 47% | 50% |  | 48% | 47% |  | 45% | 55% |  | 47% | 51% | 44% | 50% |  |
| Asset quartile |  |  | 0.002 |  |  | <0.001 |  |  | 0.03 | NR |  |  |  |  |
| Asset quartile 1 (worse off) | 27% | 15% |  | 32% | 11% |  | 26% | 23% |  |  |  |  |  |  |
| Asset quartile 2 | 23% | 31% |  | 29% | 17% |  | 23% | 27% |  |  |  |  |  |  |
| Asset quartile 3 | 24% | 29% |  | 24% | 26% |  | 23% | 29% |  |  |  |  |  |  |
| Asset quartile 4 (better off) | 25% | 23% |  | 15% | 45% |  | 27% | 19% |  |  |  |  |  |  |
| Missing | 0% | 2% |  | 0% | 1% |  | 0% | 1% |  |  |  |  |  |  |
| Household food insecurity |  |  | 0.17 |  |  | <0.001 |  |  | 0.71 |  |  |  |  | <0.001 |
| Some level of household food insecurity | 69% | 76% |  | 65% | 83% |  | 70% | 71% |  | 37% | 71% | 83% | 92% |  |
| Household is food secure | 31% | 24% |  | 35% | 17% |  | 30% | 29% |  | 63% | 29% | 17% | 8% |  |
| Water source |  |  | 0.12 |  |  | 0.005 |  |  | 0.24 |  |  |  |  | <0.001 |
| Own pump/well/tap/borehole | 31% | 24% |  | 33% | 22% |  | 28% | 32% |  | 60% | 33% | 16% | 8% |  |
| Public/neighbours well, pump or tap | 69% | 76% |  | 67% | 78% |  | 72% | 68% |  | 40% | 67% | 84% | 92% |  |
| Toilet use |  |  | 0.28 |  |  | <0.001 |  |  | 0.26 |  |  |  |  | <0.001 |
| No open defecation | 22% | 26% |  | 13% | 42% |  | 24% | 19% |  | 1% | 1% | 22% | 66% |  |
| Open defecation | 78% | 74% |  | 87% | 58% |  | 76% | 81% |  | 99% | 99% | 78% | 34% |  |
| Older siblings |  |  | 0.83 |  |  | 0.47 |  |  | 0.88 |  |  |  |  | 0.73 |
| No older siblings | 31% | 32% |  | 30% | 33% |  | 31% | 30% |  | 30% | 33% | 33% | 28% |  |
| Has older siblings | 69% | 68% |  | 70% | 67% |  | 69% | 70% |  | 70% | 67% | 67% | 72% |  |
| Birthweight |  |  | <0.001 |  |  | 0.22 | NR |  |  |  |  |  |  | 0.038 |
| Normal birthweight (≥ 2500g) | 73% | 50% |  | 68% | 70% |  |  |  |  | 71% | 64% | 64% | 75% |  |
| Low birthweight (<2500g) | 26% | 49% |  | 31% | 30% |  |  |  |  | 29% | 33% | 36% | 24% |  |
| Missing | 1% | 1% |  | 1% | 0% |  |  |  |  | 0% | 3% | 0% | 1% |  |
| Maternal education |  |  | 0.023 | NR |  |  |  |  | 0.78 |  |  |  |  | <0.001 |
| No education | 69% | 59% |  |  |  |  | 66% | 68% |  | 86% | 77% | 65% | 41% |  |
| Some level of education | 31% | 41% |  |  |  |  | 34% | 32% |  | 14% | 23% | 35% | 59% |  |
| Birth-to-pregnancy interval |  |  | <0.001 |  |  | <0.001 |  |  | <0.001 |  |  |  |  | 0.093 |
| Primigravida | 12% | 82% |  | 20% | 39% |  | 20% | 41% |  | 19% | 23% | 31% | 32% |  |
| ≥24 months | 36% | 7% |  | 34% | 22% |  | 35% | 21% |  | 34% | 35% | 23% | 29% |  |
| <24 months | 37% | 2% |  | 31% | 27% |  | 32% | 24% |  | 35% | 29% | 30% | 25% |  |
| End date of previous pregnancy unknown | 15% | 9% |  | 15% | 12% |  | 14% | 15% |  | 12% | 13% | 15% | 15% |  |
| Maternal age at birth | NR |  |  |  |  | 0.023 |  |  | <0.001 |  |  |  |  | 0.025 |
| Mother is >19 years |  |  |  | 82% | 74% |  | 85% | 68% |  | 87% | 75% | 76% | 81% |  |
| Adolescent mother (≤19y) |  |  |  | 18% | 26% |  | 15% | 32% |  | 13% | 25% | 24% | 19% |  |
| Mother ate less, same, or more in the last trimester of pregnancy |  |  | 0.24 |  |  | 0.62 |  |  | 0.86 |  |  |  |  | 0.41 |
| Ate less | 36% | 36% |  | 37% | 33% |  | 36% | 38% |  | 41% | 35% | 33% | 37% |  |
| Ate the same | 58% | 54% |  | 56% | 59% |  | 57% | 55% |  | 50% | 57% | 63% | 57% |  |
| Ate more | 6% | 11% |  | 7% | 8% |  | 7% | 7% |  | 9% | 8% | 5% | 7% |  |
| Breastfeeding within the first hour after birth |  |  | 0.027 |  |  | <0.001 |  |  | 0.75 |  |  |  |  | 0.003 |
| Child was breastfed more than one hour after birth | 75% | 65% |  | 78% | 63% |  | 72% | 74% |  | 82% | 77% | 68% | 65% |  |
| Child was breastfed within one hour after birth | 25% | 35% |  | 22% | 37% |  | 28% | 26% |  | 18% | 23% | 32% | 35% |  |
| Discard colostrum |  |  | 0.49 |  |  | 0.054 |  |  | 0.84 |  |  |  |  | 0.72 |
| Colostrum was not discarded | 77% | 79% |  | 75% | 83% |  | 78% | 77% |  | 73% | 77% | 79% | 79% |  |
| Colostrum was discarded | 23% | 20% |  | 25% | 17% |  | 22% | 22% |  | 26% | 23% | 21% | 21% |  |
| Missing | 0% | 1% |  | 0% | 1% |  | 0% | 1% |  | 1% | 0% | 0% | 1% |  |
| Maternal height (in cm)^1^ | 151 (5) | 151 (6) | 0.32 | 151 (5) | 152 (5) | 0.058 | 151 (6) | 150 (5) | <0.001 | 149 (5) | 151 (6) | 151 (6) | 152 (5) | <0.001 |

^1^ *P*-value from t-test
