# Supplementary material for: Determinants of Infant Growth in a Birth Cohort in the Nepal Plains
Source: Matern Child Nutr. 2025 Feb 26;21(3):e70004. doi: 10.1111/mcn.70004 (PMC12150145; doi:10.1111/mcn.70004)
Supplement: Supplementary file 4 — Supporting information. [file MCN-21-e70004-s004.docx]

Supplementary Table 4.1: Crude and adjusted coefficients for length-for-age *z*-score from birth to six months, from random effect models.

|  | Model 1^1^ |  | Model 2^2^ |  | Model 3^3^ |  |
| --- | --- | --- | --- | --- | --- | --- |
| Factor | Coeff (95% CI) | *p* | Coeff (95% CI) | *p* | Coeff (95% CI) | *p* |
| Intercept | -1.06 (-1.14, -0.97) | 0.00 | -0.74 (-0.92, -0.57) | 0.00 | 6.86 (4.96, 8.76) | 0.00 |
| Season of measurement |  |  |  |  |  |  |
| Spring | Ref |  | Ref |  | Ref |  |
| Monsoon | 0.12 (-0.03, 0.27) | 0.12 | 0.08 (-0.06, 0.23) | 0.27 | 0.08 (-0.06, 0.23) | 0.13 |
| Autumn | 0.03 (-0.14, 0.20) | 0.74 | -0.02 (-0.18, 0.15) | 0.86 | -0.01 (-0.18, 0.15) | 0.45 |
| Winter | -0.12 (-0.31, 0.07) | 0.22 | -0.16 (-0.34, 0.03) | 0.10 | -0.15 (-0.34, 0.03) | 0.05 |
| Child age |  |  |  |  |  |  |
| spline(age in months)_1 | -0.14 (-0.21, -0.07) | 0.00 | -0.22 (-0.35, -0.10) | 0.00 | -0.22 (-0.34, -0.09) | 0.00 |
| spline(age in months)_2 | 0.16 (0.07, 0.26) | 0.00 | 0.15 (-0.02, 0.31) | 0.08 | 0.14 (-0.03, 0.30) | 0.05 |
| spline(age in months)_3 | -0.56 (-0.61, -0.52) | 0.00 | -0.53 (-0.65, -0.40) | 0.00 | -0.53 (-0.65, -0.40) | 0.00 |
| Child sex |  |  |  |  |  |  |
| boy | Ref |  |  |  |  |  |
| girl | 0.01 (-0.15, 0.16) | 0.95 | - |  |  |  |
| Home environment |  |  |  |  |  |  |
| Asset quartile |  |  |  |  |  |  |
| Asset quartile 1 (worse off) | Ref |  |  |  |  |  |
| Asset quartile 2 | 0.11 (-0.11, 0.33) | 0.32 | - |  | - |  |
| Asset quartile 3 | 0.30 (0.09, 0.51) | 0.01 | - |  | - |  |
| Asset quartile 4 (better off) | 0.31 (0.09, 0.52) | 0.01 | - |  | - |  |
| Household food insecurity |  |  |  |  |  |  |
| Some level of household food insecurity | Ref |  |  |  |  |  |
| Household is food secure | 0.21 (0.04, 0.38) | 0.01 | - |  | - |  |
| Water source |  |  |  |  |  |  |
| Own pump/well/tap/borehole | Ref |  |  |  |  |  |
| Public/neighbours well, pump or tap | -0.09 (-0.26, 0.08) | 0.31 | - |  | - |  |
| Toilet use |  |  |  |  |  |  |
| No open defecation | Ref |  |  |  |  |  |
| Open defecation | -0.23 (-0.42, -0.05) | 0.01 | - |  | - |  |
| Number of older siblings |  |  |  |  |  |  |
| None | Ref |  |  |  |  |  |
| One | -0.46 (-0.71, -0.22) | 0.00 | - |  | - |  |
| Two or more | 0.08 (-0.11, 0.27) | 0.41 | - |  | - |  |
| Interaction Age : Number of older siblings |  |  |  |  |  |  |
| spline(age in months)_1:One | -0.14 (-0.34, 0.06) | 0.18 | - |  | - |  |
| spline(age in months)_2:One | 0.26 (-0.01, 0.53) | 0.06 | - |  | - |  |
| spline(age in months)_3:One | 0.37 (0.24, 0.51) | 0.00 | - |  | - |  |
| spline(age in months)_1:Two or more | -0.43 (-0.59, -0.27) | 0.00 | - |  | - |  |
| spline(age in months)_2:Two or more | -0.18 (-0.39, 0.03) | 0.10 | - |  | - |  |
| spline(age in months)_3:Two or more | -0.06 (-0.17, 0.04) | 0.22 | - |  | - |  |
| Maternal factors |  |  |  |  |  |  |
| Birthweight |  |  |  |  |  |  |
| Normal birthweight (≥ 2500g) | Ref |  | Ref |  | Ref |  |
| Low birthweight (<2500g) | -1.24 (-1.40, -1.08) | 0.00 | -1.21 (-1.38, -1.05) | 0.00 | -1.13 (-1.29, -0.96) | 0.00 |
| Interaction Age : low birthweight |  |  |  |  |  |  |
| spline(age in months)_1:low birthweight | 0.27 (0.12, 0.42) | 0.00 | 0.25 (0.11, 0.40) | 0.00 | 0.25 (0.10, 0.40) | 0.00 |
| spline(age in months)_2:low birthweight | 0.44 (0.24, 0.63) | 0.00 | 0.43 (0.23, 0.62) | 0.00 | 0.42 (0.22, 0.62) | 0.00 |
| spline(age in months)_3:low birthweight | 0.51 (0.41, 0.60) | 0.00 | 0.48 (0.38, 0.58) | 0.00 | 0.47 (0.38, 0.57) | 0.00 |
| Maternal education |  |  |  |  |  |  |
| No education | Ref |  | Ref |  | Ref |  |
| Some level of education | 0.07 (-0.11, 0.25) | 0.48 | 0.05 (-0.11, 0.21) | 0.51 | 0.01 (-0.14, 0.17) | 0.44 |
| Interaction Age : Mat. education | 0.00 (0.00, 0.00) |  |  |  |  |  |
| spline(age in months)_1:  Some level of education | 0.23 (0.08, 0.38) | 0.00 | 0.24 (0.10, 0.38) | 0.00 | 0.23 (0.09, 0.38) | 0.00 |
| spline(age in months)_2:  Some level of education | 0.38 (0.18, 0.58) | 0.00 | 0.36 (0.16, 0.55) | 0.00 | 0.36 (0.17, 0.56) | 0.00 |
| spline(age in months)_3:  Some level of education | 0.18 (0.08, 0.28) | 0.00 | 0.16 (0.07, 0.26) | 0.00 | 0.16 (0.07, 0.26) | 0.00 |
| Birth-to-pregnancy interval |  |  |  |  |  |  |
| ≥24 months | Ref |  |  |  |  |  |
| <24 months | 0.21 (0.00, 0.43) | 0.05 | - |  | - |  |
| End date of previous pregnancy unknown | -0.25 (-0.52, 0.03) | 0.08 | - |  | - |  |
| Primigravida | -0.41 (-0.63, -0.18) | 0.00 | - |  | - |  |
| Interaction Age : Birth-to-pregnancy interval |  |  |  |  |  |  |
| spline(age in months)_1:<24 mths | -0.16 (-0.33, 0.01) | 0.07 | - |  | - |  |
| spline(age in months)_2:<24 mths | -0.27 (-0.50, -0.04) | 0.02 | - |  | - |  |
| spline(age in months)_3:<24 mths | -0.28 (-0.39, -0.16) | 0.00 | - |  | - |  |
| spline(age in months)_1:  End date of previous pregnancy unknown | -0.06 (-0.29, 0.17) | 0.60 | - |  | - |  |
| spline(age in months)_2:  End date of previous pregnancy unknown | -0.17 (-0.48, 0.14) | 0.27 | - |  | - |  |
| spline(age in months)_3:  End date of previous pregnancy unknown | 0.11 (-0.04, 0.26) | 0.14 | - |  | - |  |
| spline(age in months)_1:Primigravida | 0.19 (0.01, 0.38) | 0.04 | - |  | - |  |
| spline(age in months)_2:Primigravida | 0.37 (0.12, 0.61) | 0.00 | - |  | - |  |
| spline(age in months)_3:Primigravida | 0.31 (0.19, 0.44) | 0.00 | - |  | - |  |
| Maternal age at birth |  |  |  |  |  |  |
| Mother is >19 years | Ref |  | Ref |  | Ref |  |
| Adolescent mother (≤19y) | -0.45 (-0.67, -0.24) | 0.00 | -0.22 (-0.41, -0.03) | 0.03 | -0.26 (-0.44, -0.07) | 0.00 |
| Interaction Age : Adolescent mother |  |  |  |  |  |  |
| spline(age in months)_1:  Adolescent mother | 0.21 (0.03, 0.39) | 0.02 | 0.13 (-0.05, 0.30) | 0.16 | 0.13 (-0.05, 0.30) | 0.08 |
| spline(age in months)_2:  Adolescent mother | 0.23 (0.00, 0.47) | 0.05 | 0.08 (-0.15, 0.32) | 0.47 | 0.10 (-0.13, 0.33) | 0.21 |
| spline(age in months)_3:  Adolescent mother | 0.28 (0.16, 0.40) | 0.00 | 0.15 (0.04, 0.27) | 0.01 | 0.15 (0.04, 0.27) | 0.00 |
| Maternal absence and feeding arrangement * |  |  |  |  |  |  |
| Doesn't work outside, takes break to feed the baby or takes baby with her | Ref |  |  |  |  |  |
| Baby is with another carer | -0.27 (-2.96, 2.41) | 0.84 | - |  | - |  |
| No feeding arrangement | 0.46 (-1.36, 2.28) | 0.62 | - |  | - |  |
| Interaction Age : Maternal absence and feeding arrangement |  |  |  |  |  |  |
| spline(age in months)_1:  Baby is with another carer | -0.07 (-1.35, 1.22) | 0.92 | - |  | - |  |
| spline(age in months)_2:  Baby is with another carer | 0.67 (-4.66, 6.00) | 0.81 | - |  | - |  |
| spline(age in months)_3:  Baby is with another carer | 0.01 (-1.33, 1.34) | 0.99 | - |  | - |  |
| spline(age in months)_1:  No feeding arrangement | -0.31 (-1.19, 0.57) | 0.48 | - |  | - |  |
| spline(age in months)_2:  No feeding arrangement | -0.73 (-4.33, 2.87) | 0.69 | - |  | - |  |
| spline(age in months)_3:  No feeding arrangement | -0.34 (-1.24, 0.56) | 0.46 | - |  | - |  |
| Mother ate less, same, or more in the last trimester of pregnancy |  |  |  |  |  |  |
| Ate less | Ref |  |  |  |  |  |
| Ate the same | 0.14 (-0.02, 0.30) | 0.10 | - |  | - |  |
| Ate more | 0.29 (-0.03, 0.61) | 0.07 | - |  | - |  |
| Maternal height (in cm) |  |  |  |  | 0.04 (0.02, 0.05) | 0.00 |
| Infection |  |  |  |  |  |  |
| Diarrhoea |  |  |  |  |  |  |
| No diarrhoea in the previous two weeks | Ref |  |  |  |  |  |
| Child had diarrhoea in the previous two weeks | -0.05 (-0.12, 0.02) | 0.19 | - |  | - |  |
| Symptom of chest infection** |  |  |  |  |  |  |
| No cough with rapid breathing | Ref |  | Ref |  | Ref |  |
| Cough and rapid breathing | -0.10 (-0.15, -0.05) | 0.00 | -0.10 (-0.14, -0.05) | 0.00 | -0.10 (-0.15, -0.05) | 0.00 |
| Breastfeeding practices |  |  |  |  |  |  |
| Breastfeeding within the first hour after birth |  |  |  |  |  |  |
| Child was breastfed more than one hour after birth | Ref |  |  |  |  |  |
| Child was breastfed within one hour after birth | 0.23 (0.06, 0.40) | 0.01 | - |  | - |  |
| Exclusive breastfeeding |  |  |  |  |  |  |
| Child was not exclusively breastfed in the first six months | Ref |  |  |  |  |  |
| Child was exclusively breastfed in the first six months | 0.02 (-0.03, 0.07) | 0.49 | - |  | - |  |
| Discard colostrum |  |  |  |  |  |  |
| Colostrum was not discarded | Ref |  |  |  |  |  |
| Colostrum was discarded | 0.01 (-0.18, 0.19) | 0.93 | - |  |  |  |

^1^ Unadjusted (univariable) mixed-effects linear regression models between the respective potential determinant and the outcome length-for-age *z*-score, with a random effect on the intercept. The dataset contains all observations with complete data in those variables that were selected as potential determinants of infant growth (*n*=3829).

^2^ Multivariable mixed-effects linear regression model adjusted for those factors that were identified as relevant in Models 1, using BIC as criterion for goodness of fit. The final model contains child age, low birthweight, maternal education, maternal age at birth, chest infection, and season of measurement as fixed effects, and a random effect on the intercept (*n*=3907).

^3^ Multivariable mixed-effects linear regression models using the dataset with imputed covariates, adjusted for the same factors as Model 2 and additionally for maternal height (*n*=4216).

*In the three months preceding the interview. **In the two weeks preceding the interview.

Supplementary Table 4.2: Crude and adjusted coefficients for length-for-age *z*-score from 7 to 24 months, from random effect models.

|  | Model 1^1^ |  | Model 2^2^ |  | Model 3^3^ |  |
| --- | --- | --- | --- | --- | --- | --- |
| Factor | Coeff (95% CI) | *p* | Coeff (95% CI) | *p* | Coeff (95% CI) | *p* |
| Intercept | -1.35 (-1.43, -1.27) | 0.00 | -1.24 (-1.43, -1.06) | 0.00 | 10.03 (8.14, 11.92) | 0.00 |
| Season of measurement |  |  |  |  |  |  |
| Spring | Ref |  | Ref |  | Ref |  |
| Monsoon | 0.03 (0.01, 0.06) | 0.00 | 0.03 (0.01, 0.06) | 0.00 | 0.03 (0.01, 0.06) | 0.00 |
| Autumn | 0.00 (-0.02, 0.03) | 0.73 | 0.01 (-0.02, 0.04) | 0.48 | 0.01 (-0.02, 0.03) | 0.28 |
| Winter | -0.07 (-0.09, -0.05) | 0.00 | -0.06 (-0.08, -0.04) | 0.00 | -0.06 (-0.08, -0.04) | 0.00 |
| Child age |  |  |  |  |  |  |
| spline(age in months)_1 | -0.80 (-0.82, -0.77) | 0.00 | -0.90 (-1.00, -0.79) | 0.00 | -0.75 (-0.80, -0.71) | 0.00 |
| spline(age in months)_2 | -0.39 (-0.42, -0.36) | 0.00 | -0.52 (-0.63, -0.42) | 0.00 | -0.41 (-0.46, -0.36) | 0.00 |
| spline(age in months)_3 | -1.22 (-1.28, -1.16) | 0.00 | -1.63 (-1.90, -1.36) | 0.00 | -1.41 (-1.51, -1.30) | 0.00 |
| spline(age in months)_4 | -0.45 (-0.48, -0.41) | 0.00 | -0.49 (-0.58, -0.39) | 0.00 | -0.52 (-0.57, -0.47) | 0.00 |
| Child sex |  |  |  |  |  |  |
| boy | Ref |  | - |  |  |  |
| girl | 0.02 (-0.13, 0.18) | 0.76 | - |  | - |  |
| Home environment |  |  |  |  |  |  |
| Asset quartile |  |  |  |  |  |  |
| Asset quartile 1 (worse off) | Ref |  |  |  |  |  |
| Asset quartile 2 | 0.22 (0.01, 0.44) | 0.04 | - |  | - |  |
| Asset quartile 3 | 0.32 (0.10, 0.53) | 0.00 | - |  | - |  |
| Asset quartile 4 (better off) | 0.32 (0.11, 0.53) | 0.00 | - |  | - |  |
| Household food insecurity |  |  |  |  |  |  |
| Some level of household food insecurity | Ref |  | Ref |  | Ref |  |
| Household is food secure | 0.29 (0.12, 0.45) | 0.00 | 0.23 (0.07, 0.39) | 0.00 | 0.16 (0.01, 0.31) | 0.02 |
| Water source |  |  |  |  |  |  |
| Own pump/well/tap/borehole | Ref |  | Ref |  | Ref |  |
| Public/neighbours well, pump or tap | -0.14 (-0.31, 0.04) | 0.12 | -0.02 (-0.19, 0.14) | 0.79 | -0.01 (-0.16, 0.15) | 0.47 |
| Interaction Age : Water source |  |  |  |  |  |  |
| spline(age in months)_1:  Public/neighbours well, pump or tap | -0.08 (-0.13, -0.02) | 0.01 | -0.06 (-0.12, -0.01) | 0.02 | -0.07 (-0.12, -0.02) | 0.01 |
| spline(age in months)_2:  Public/neighbours well, pump or tap | -0.15 (-0.22, -0.08) | 0.00 | -0.14 (-0.21, -0.07) | 0.00 | -0.14 (-0.21, -0.07) | 0.00 |
| spline(age in months)_3:  Public/neighbours well, pump or tap | -0.09 (-0.22, 0.04) | 0.18 | -0.09 (-0.22, 0.04) | 0.19 | -0.10 (-0.23, 0.03) | 0.06 |
| spline(age in months)_4:  Public/neighbours well, pump or tap | -0.07 (-0.15, 0.00) | 0.05 | -0.08 (-0.16, -0.01) | 0.02 | -0.09 (-0.16, -0.01) | 0.01 |
| Toilet use |  |  |  |  |  |  |
| No open defecation | Ref |  |  |  |  |  |
| Open defecation | -0.21 (-0.39, -0.02) | 0.03 | - |  | - |  |
| Any older siblings |  |  |  |  |  |  |
| No older siblings | Ref |  |  |  |  |  |
| Has older siblings | 0.10 (-0.07, 0.27) | 0.26 | - |  | - |  |
| Interaction Age : Older siblings |  |  |  |  |  |  |
| spline(age in months)_1:  Has older siblings | -0.16 (-0.21, -0.10) | 0.00 | - |  | - |  |
| spline(age in months)_2:  Has older siblings | 0.05 (-0.02, 0.12) | 0.14 | - |  | - |  |
| spline(age in months)_3:  Has older siblings | -0.34 (-0.47, -0.21) | 0.00 | - |  | - |  |
| spline(age in months)_4:  Has older siblings | -0.04 (-0.12, 0.03) | 0.25 | - |  | - |  |
| Maternal factors |  |  |  |  |  |  |
| Birthweight |  |  |  |  |  |  |
| Normal birthweight (≥ 2500g) | Ref |  | Ref |  | Ref |  |
| Low birthweight (<2500g) | -0.75 (-0.91, -0.58) | 0.00 | -0.75 (-0.91, -0.59) | 0.00 | -0.63 (-0.78, -0.48) | 0.00 |
| Interaction Age : low birthweight |  |  |  |  |  |  |
| spline(age in months)_1:  low birthweight | 0.20 (0.14, 0.25) | 0.00 | 0.18 (0.13, 0.24) | 0.00 | 0.18 (0.13, 0.23) | 0.00 |
| spline(age in months)_2:  low birthweight | 0.16 (0.08, 0.23) | 0.00 | 0.16 (0.09, 0.23) | 0.00 | 0.15 (0.09, 0.22) | 0.00 |
| spline(age in months)_3:  low birthweight | 0.23 (0.10, 0.36) | 0.00 | 0.22 (0.09, 0.35) | 0.00 | 0.22 (0.09, 0.35) | 0.00 |
| spline(age in months)_4:  low birthweight | 0.29 (0.22, 0.36) | 0.00 | 0.29 (0.22, 0.36) | 0.00 | 0.28 (0.21, 0.36) | 0.00 |
| Maternal education |  |  |  |  |  |  |
| No education | Ref |  | Ref |  | Ref |  |
| Some level of education | 0.30 (0.13, 0.46) | 0.00 | 0.22 (0.07, 0.38) | 0.00 | 0.18 (0.03, 0.32) | 0.01 |
| Birth-to -pregnancy interval |  |  |  |  |  |  |
| ≥24 months | Ref |  |  |  |  |  |
| <24 months | -0.16 (-0.36, 0.04) | 0.12 | - |  | - |  |
| End date of previous pregnancy unknown | -0.27 (-0.52, -0.02) | 0.04 | - |  | - |  |
| Primigravida | 0.05 (-0.16, 0.25) | 0.64 | - |  | - |  |
| Maternal age at birth |  |  |  |  |  |  |
| Mother is >19 years | Ref |  |  |  |  |  |
| Adolescent mother (≤19y) | -0.02 (-0.21, 0.18) | 0.85 | - |  | - |  |
| Maternal absence and feeding arrangement * |  |  |  |  |  |  |
| Doesn't work outside, takes break to feed the baby or takes baby with her | Ref |  | Ref |  | Ref |  |
| Baby is with another carer | 0.14 (0.03, 0.25) | 0.01 | 0.09 (-0.02, 0.19) | 0.10 | 0.09 (-0.01, 0.19) | 0.04 |
| No feeding arrangement | 0.13 (0.05, 0.20) | 0.00 | 0.02 (-0.10, 0.14) | 0.72 | 0.11 (0.03, 0.18) | 0.00 |
| Interaction Age : Maternal absence and feeding arrangement |  |  |  |  |  |  |
| spline(age in months)_1:  Baby is with another carer | -0.19 (-0.30, -0.08) | 0.00 | -0.14 (-0.24, -0.04) | 0.01 | -0.14 (-0.24, -0.03) | 0.00 |
| spline(age in months)_2:  Baby is with another carer | -0.14 (-0.25, -0.03) | 0.01 | -0.11 (-0.22, 0.00) | 0.04 | -0.11 (-0.22, 0.00) | 0.02 |
| spline(age in months)_3:  Baby is with another carer | -0.33 (-0.61, -0.05) | 0.02 | -0.22 (-0.48, 0.05) | 0.11 | -0.22 (-0.48, 0.05) | 0.05 |
| spline(age in months)_4:  Baby is with another carer | 0.01 (-0.09, 0.11) | 0.88 | 0.04 (-0.06, 0.14) | 0.46 | 0.04 (-0.06, 0.14) | 0.21 |
| spline(age in months)_1:  No feeding arrangement | -0.36 (-0.45, -0.28) | 0.00 | -0.20 (-0.32, -0.07) | 0.00 | -0.33 (-0.42, -0.25) | 0.00 |
| spline(age in months)_2:  No feeding arrangement | -0.13 (-0.23, -0.02) | 0.02 | 0.03 (-0.11, 0.17) | 0.12 | -0.08 (-0.18, 0.02) | 0.69 |
| spline(age in months)_3:  No feeding arrangement | -0.35 (-0.56, -0.14) | 0.00 | -0.04 (-0.36, 0.28) | 0.01 | -0.25 (-0.45, -0.04) | 0.82 |
| spline(age in months)_4:  No feeding arrangement | -0.22 (-0.33, -0.11) | 0.00 | -0.21 (-0.34, -0.07) | 0.00 | -0.17 (-0.28, -0.06) | 0.00 |
| Mother ate less, same, or more in the last trimester of pregnancy |  |  |  |  |  |  |
| Ate less | Ref |  |  |  |  |  |
| Ate the same | -0.07 (-0.24, 0.09) | 0.38 | - |  | - |  |
| Ate more | -0.06 (-0.39, 0.26) | 0.70 | - |  | - |  |
| Maternal height (in cm) | - |  | - |  | 0.06 (0.05, 0.07) | 0.00 |
| Infection |  |  |  |  |  |  |
| Diarrhoea ** |  |  |  |  |  |  |
| No diarrhoea | Ref |  |  |  |  |  |
| Child had diarrhoea | -0.01 (-0.03, 0.00) | 0.10 | - |  | - |  |
| Symptom of chest infection ** |  |  | - |  |  |  |
| No cough with rapid breathing | Ref |  |  |  |  |  |
| Cough and rapid breathing | -0.02 (-0.04, 0.00) | 0.08 | - |  | - |  |
| Breastfeeding practices |  |  |  |  |  |  |
| Breastfeeding within the first hour after birth |  |  |  |  |  |  |
| Child was breastfed more than one hour after birth |  |  |  |  |  |  |
| Child was breastfed within one hour after birth | - |  | - |  | - |  |
| Exclusive breastfeeding |  |  |  |  |  |  |
| Child was not exclusively breastfed in the first six months |  |  |  |  |  |  |
| Child was exclusively breastfed in the first six months | - |  | - |  | - |  |
| Discard colostrum |  |  |  |  |  |  |
| Colostrum was not discarded | Ref |  |  |  |  |  |
| Colostrum was discarded | 0.04 (-0.15, 0.22) | 0.69 | - |  | - |  |
| Complementary feeding practices |  |  |  |  |  |  |
| Child dietary diversity *** |  |  |  |  |  |  |
| No minimum dietary diversity (<4 of 7 food groups) | Ref |  | Ref |  | Ref |  |
| Minimum dietary diversity (≥4 of 7 food groups) | 0.04 (0.02, 0.06) | 0.00 | 0.03 (0.01, 0.05) | 0.00 | 0.03 (0.01, 0.05) | 0.00 |
| Meal frequency *** |  |  |  |  |  |  |
| No minimum meal frequency | Ref |  |  |  |  |  |
| Minimum meal frequency | -0.02 (-0.03, 0.00) | 0.05 | - |  | - |  |
| Continued breastfeeding |  |  |  |  |  |  |
| Child is not breastfed anymore | Ref |  |  |  |  |  |
| Child is still breastfed | 0.05 (0.01, 0.08) | 0.02 | - |  | - |  |

^1^ Unadjusted (univariable) mixed-effects linear regression models between the respective potential determinant and the outcome length-for-age *z*-score, with a random effect on the intercept. The dataset contains all observations with complete data in those variables that were selected as potential determinants of infant growth (*n*=8411).

^2^ Multivariable mixed effects linear regression model adjusted for those factors that were identified as relevant in Models 1, using BIC as criterion for goodness of fit. The final model contains child age, low birthweight, maternal education, maternal age at birth, chest infection, and season of measurement as fixed effects, and a random effect on the intercept (*n*=8920).

^3^ Multivariable mixed effects linear regression models using the dataset with imputed covariates, adjusted for the same factors as Model 2 and additionally for maternal height (*n*=9511).

*In the three months preceding the interview. **In the two weeks preceding the interview. ***In the 24 hours preceding the interview
